# Resmetirom for nonalcoholic fatty liver disease: a randomized, double-blind, placebo-controlled phase 3 trial

**DOI:** 10.1038/s41591-023-02603-1

**Published:** 2023-10-16

**Authors:** Stephen A. Harrison, Rebecca Taub, Guy W. Neff, K. Jean Lucas, Dominic Labriola, Sam E. Moussa, Naim Alkhouri, Mustafa R. Bashir

**Affiliations:** 1 Pinnacle Clinical Research, San Antonio, TX USA; 2 Madrigal Pharmaceuticals, Conshohocken, PA USA; 3 Covenant Metabolic Specialists, Sarasota, FL USA; 4 Lucas Research, Morehead City, NC USA; 5 University of Arizona for Medical Sciences, Tucson, AZ USA; 6 Arizona Liver Health, Tucson, AZ USA; 7 Duke University Medical Center, Durham, NC USA

**Keywords:** Endocrine system and metabolic diseases, Drug development

## Abstract

Nonalcoholic steatohepatitis (NASH) is a progressive liver disease with no approved treatment. MAESTRO-NAFLD-1 was a 52-week randomized, double-blind, placebo-controlled phase 3 trial evaluating the safety of resmetirom in adults with nonalcoholic fatty liver disease and presumed NASH. Patients were randomized to three double-blind arms (100 mg resmetirom (*n* = 325), 80 mg resmetirom (*n* = 327) or placebo (*n* = 320)) or open-label 100 mg resmetirom (*n* = 171). The primary end point was incidence of treatment-emergent adverse events (TEAEs) over 52 weeks and key secondary end points were LDL-C, apoB, triglycerides (over 24 weeks), hepatic fat (over 16 and 52 weeks) and liver stiffness (over 52 weeks). Resmetirom was safe and well tolerated. TEAEs occurred in 86.5% (open-label 100 mg resmetirom), 86.1% (100 mg resmetirom), 88.4% (80 mg resmetirom) and 81.8% (placebo) of patients. TEAEs in excess of placebo included diarrhea and nausea at the initiation of treatment. Key secondary end points included least square means difference from placebo at 80 mg, 100 mg resmetirom: LDL-C (−11.1%, −12.6%), apoB (−15.6%, −18.0%), triglycerides (−15.4%, −20.4%), 16-week hepatic fat (−34.9%, −38.6%), (*P* < 0.0001) and liver stiffness (−1.02, −1.70) and 52-week hepatic fat (−28.8, −33.9). These findings demonstrate resmetirom was safe and well tolerated in adults with presumed NASH, supporting a role for further clinical development. (ClinicalTrials.gov identifier NCT04197479).

## Abstract (web summary)

In the phase 3 MAESTRO-NAFLD-1 trial, the liver-targeted thyroid hormone receptor-β selective agonist resmetirom was well tolerated and improved liver biomarkers in adults with nonalcoholic fatty liver disease.

## Main

Nonalcoholic fatty liver disease (NAFLD) is associated with metabolic dysregulation and is commonly identified in individuals with obesity, type 2 diabetes and dyslipidemia^1^. Overall, the global prevalence of NAFLD is estimated to be approximately 25% (refs. ^2–4^), with higher prevalence among patients with comorbid conditions such as obesity and type 2 diabetes^5,6^. In addition, the prevalence of NAFLD is projected to increase in subsequent decades with the rising prevalence of obesity^7,8^. In general, 25% of patients with NAFLD have NASH, defined as the presence of ≥5% hepatic fat (steatosis) in combination with hepatocyte injury (ballooning) and inflammation^9–11^. In a subset of patients, NASH will further progress to advanced fibrosis and cirrhosis (which can necessitate liver transplantation), portal hypertension, hepatocellular carcinoma and death^4,8^. There are currently no approved treatments for NASH.

At present, a liver biopsy is needed to definitively diagnose NASH; however, liver biopsy is an invasive procedure with associated morbidity. As such, noninvasive tests (biomarkers and/or imaging techniques) that can replace serial liver biopsies in (1) identifying patients with NASH and (2) monitoring treatment response (in the setting of an approved therapy) are urgently needed. Magnetic resonance imaging-proton density fat fraction (MRI-PDFF) is an accurate imaging technique that quantifies hepatic fat content. A systematic review and meta-analysis performed by Stine et al. showed that adults with NASH who achieved a ≥30% reduction from baseline in hepatic fat (measured by MRI-PDFF) had greater odds of achieving NASH reduction and resolution, suggesting this threshold could be used as a marker for improvement in NASH^12^.

Thyroid hormone receptor (THR)-β is responsible for regulating metabolic pathways in the liver and is frequently impaired in NASH^13^. Patients with NASH have reduced levels of thyroid hormone activity in the liver with resultant impaired hepatic function. Resmetirom is an oral, once-daily, liver-targeted THR-β selective agonist in clinical development for the treatment of NASH. In a randomized, double-blind (DB), placebo-controlled phase 2 serial liver biopsy trial in adults with biopsy-confirmed NASH, resmetirom-treated patients achieved a significantly greater relative reduction from baseline in hepatic fat (−32.9% versus −10.4%; *P* < 0.0001) (ref. ^14^). Reduction in hepatic fat of ≥30% by resmetirom was associated with an increased rate of NASH resolution (37%) as well as improvements in patient-reported outcomes (PROs)^14^. After completing the phase 2 trial, resmetirom-treated patients who rolled over into a 36-week open-label extension that used higher resmetirom doses of 80 and 100 mg once daily achieved a 50% and 64% relative reduction in hepatic fat, respectively^15^. The potential efficacy and adverse event profile of the phase 2 trials supported the selection of 80 and 100 mg resmetirom for phase 3 (ref. ^15^). MAESTRO-NAFLD-1 (NCT04951219) is one of four phase 3 trials that have been initiated (MAESTRO-NASH (NCT03900429), MAESTRO-NAFLD-1, MAESTRO-NAFLD-OLE and MAESTRO-NASH-OUTCOMES (NCT05500222)) to support an indication for the treatment of NASH with liver fibrosis.

To increase the overall size of the safety database, MAESTRO-NAFLD-1 was a randomized, DB, placebo-controlled phase 3 trial to evaluate the safety and tolerability of 80 and 100 mg resmetirom versus placebo over 52 weeks of treatment in adults with NAFLD (presumed NASH) diagnosed utilizing noninvasive biomarkers and imaging (as opposed to an invasive diagnostic liver biopsy). Primary, key secondary and secondary end points from MAESTRO-NAFLD-1 are reported here.

## Results

### Patient disposition

**Fig. 1:**
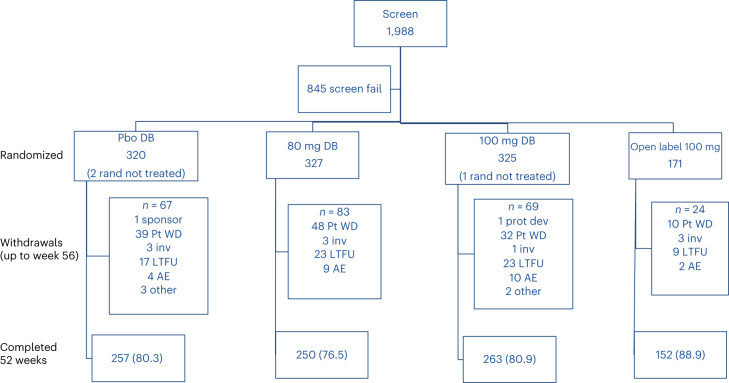
Patient composition. Pbo, placebo; rand, randomized; Pt, patient; WD, withdrew; inv, investigator decision; LFTU, lost to follow-up; AE, adverse event; prot dev, protocol deviation.

MAESTRO-NAFLD-1 was conducted between 16 December 2019 and 13 December 2021 at 80 sites in the United States. Overall, 1,988 patients were screened and 1,143 patients were randomized to the trial (Fig. 1).

Overall, 972 patients were randomized to the three DB arms (100 mg resmetirom (*n* = 325), 80 mg resmetirom (*n* = 327) or placebo (*n* = 320)) and 171 patients were randomized to the open-label (OL) 100 mg resmetirom arm. One patient in the DB 100 mg resmetirom arm and two patients in the placebo arm were randomized but did not receive the study drug (as study sites were closed due to COVID-19). As such, 969 patients randomized to DB treatment and 171 patients randomized to OL 100 mg resmetirom treatment were included in the safety population. In total, 77.4% of patients (750 out of 969) across the three DB arms completed the study, whereas 22.6% (219 out of 969) were discontinued from the trial. Of the 171 patients randomized to the OL 100 mg resmetirom arm, 89.0% (152 out of 171) completed the 52-week treatment period. The percentage of patients who were discontinued from the trial did not markedly differ among the three DB arms. Most patients discontinued the trial due to patient withdrawal (other than TEAEs) or were lost to follow-up (119 and 63 patients across the three DB arms, respectively).

Access to study drug was impacted by the COVID-19 pandemic, particularly in the DB arms where drug kit delays occurred, but not in the OL arm that utilized bottles of tablets. Largely due to COVID-19-related drug kit delays in the DB arms, 86–88% of DB patients missed study visits (inclusive of visits where no study drug was provided) and the average number of missed monthly visits in the DB arms was 2–3 over the 52-week treatment period. Only 19% of OL patients had missed study visits. Compliance was 81.2% in the OL arm and 76.4% across the three DB arms. The mean number of weeks of exposure to study drug was 47 weeks in the OL 100 mg resmetirom arm, 45 weeks in the DB 100 mg resmetirom arm, 43 weeks in the DB 80 mg resmetirom arm and 45 weeks in the placebo arm. Study drug dose adjustments (based on low free thyroxine (FT4) levels, defined as a ≥30% decrease from baseline on consecutive visits to a value of <0.7 ng dl^−1^) were infrequent; 12 (2.4%) patients had their dose reduced from 100 mg resmetirom to 80 mg and 2 (0.6%) patients had their dose reduced from 80 mg resmetirom to 60 mg.

Across the four treatment arms, demographic and baseline characteristics of the safety population were generally comparable (Table 1) with mean age, 56 years; female, 57%; white, 88%; and Hispanic, 34%. High percentages of patients across all four arms had metabolic risk factors including obesity with mean body mass index (BMI) 35 kg m^−^^2^; type 2 diabetes, 49%; dyslipidemia, 88%; and hypertension, 75%. Per protocol, patients with hypothyroidism on thyroxine doses >75 μg were enrolled in the OL 100 mg resmetirom arm (to allow for comparison with patients not on thyroxine). Patients with a diagnosis of hypothyroidism on thyroxine doses ≤75 μg were enrolled in the DB arms until the protocol was amended near the end of the randomization period to allow patients on thyroxine doses >75 μg to enroll in the DB arms. As such, 44.4% of patients in the OL 100 mg resmetirom arm were on thyroxine at baseline compared to 10.5–11.9% in the DB arms. Consistent with the higher percentage of patients on thyroxine treatment, a higher percentage of patients in the OL arm were female. Common concomitant medications across the four arms were antidiabetes drugs (such as glucagon-like peptide-1 (GLP-1) receptor agonists (RAs), metformin, pioglitazone and sodium/glucose cotransporter-2 inhibitors) and drugs to manage dyslipidemia (statins, 46%). A greater proportion of patients in the OL arm were taking GLP-1 RAs (11.7%) and SGLT2 inhibitors (10.5%) at baseline compared to the three DB arms (6.0–9.3% and 4.7–9.3%, respectively). Demographic and baseline characteristics of the modified intent-to-treat (mITT) population (which included all randomized patients who received ≥1 dose of DB study drug and had a baseline and ≥1 post-baseline measurement) are shown in Extended Data Table 1. The demographic and baseline characteristics of the safety and mITT populations were comparable.

**Table 1:** Demographic and baseline characteristics (safety population)

	Resmetirom 100 mg OL (*n* = 171)	Resmetirom 100 mg DB (*n* = 324)	Resmetirom 80 mg DB (*n* = 327)	Placebo DB (*n* = 318)
Age, years, mean (s.d.)	55.6 (11.5)	55.9 (11.7)	56.2 (11.7)	55.7 (12.1)
Sex, male, *n* (%)^a^	55 (32.2)	147 (45.4)	145 (44.3)	150 (47.2)
Race, *n* (%)				
White	151 (88.3)	287 (88.6)	290 (88.7)	282 (88.7)
Black or African American	10 (5.8)	22 (6.8)	21 (6.4)	20 (6.3)
Asian	6 (3.5)	6 (1.9)	6 (1.8)	7 (2.2)
Other^b^	3 (1.8)	7 (2.1)	8 (2.4)	7 (2.2)
Missing	1 (0.6)	2 (0.6)	2 (0.6)	2 (0.6)
Ethnicity, Hispanic or Latino, *n* (%)	52 (30.4)	108 (33.3)	108 (33.0)	120 (37.7)
BMI, kg m^−^^2^, mean (s.d.)	36.1 (6.3)	35.4 (6.4)	35.3 (5.9)	35.2 (5.8)
Type 2 diabetes, *n* (%)	83 (48.5)	156 (48.1)	160 (48.9)	159 (50.0)
Hypertension, *n* (%)	119 (69.6)	246 (75.9)	249 (76.1)	242 (76.1)
Dyslipidemia, *n* (%)	152 (88.9)	283 (87.3)	288 (88.1)	281 (88.4)
Hypothyroidism, *n* (%)^c^	76 (44.4)	34 (10.5)	39 (11.9)	35 (11.0)
ASCVD, *n* (%)	10 (5.8)	22 (6.8)	26 (8.0)	24 (7.5)
10-year ASCVD risk score, %	*n* = 140	*n* = 253	*n* = 245	*n* = 238
Mean (s.d.)	11.6 (12.5)	12.2 (11.7)	12.7 (11.5)	13.5 (12.8)
Median (Q1, Q3)	6.6 (3.4, 15.2)	8.4 (4.0, 16.2)	9.6 (3.9, 16.5)	9.4(3.7, 20.0)
FibroScan VCTE/LSM, kPa, mean (s.d.)	7.8 (3.4)	7.3 (4.1)	7.4 (4.4)	7.5 (5.5)
FibroScan CAP, dBm, mean (s.d.)	342.3 (35.6)	341.4 (34.0)	339.5 (32.9)	344.1 (34.0)
MRI-PDFF, % fat fraction, mean (s.d.)	17.9 (7.1)	18.1 (7.3)	17.7 (6.7)	17.8 (6.9)
MRE, kPa, mean (s.d.)	2.8 (0.9) *n* = 114	2.6 (0.5) *n* = 232	2.6 (0.5) *n* = 219	2.60 (0.50) *n* = 205
FIB-4, mean (s.d.)	1.0 (0.6)	1.0 (0.4)	1.0 (0.5)	1.0 (0.50)
ALT, U l^−1^, mean (s.d.)	36.9 (24.2)	36.2 (25.2)	37.1 (23.9)	37.9 (30.4)
AST, U l^−1^, mean (s.d.)	26.4 (15.3)	24.9 (12.4)	25.3 (13.3)	26.4 (16.4)
GGT, U l^−1^				
Mean (s.d.)	46.9 (55.0)	41.5 (31.8)	46.1 (41.0)	49.9 (62.1)
Median (Q1, Q3)	30 (22, 47)	32 (22, 49)	33 (25, 49)	33(24, 52)
ALP, U l^−1^, mean (s.d.)	72.8 (23.8)	70.8 (22.3)	71.6 (23.8)	71.3 (24.8)
Platelets, 10 l^−1^, mean (s.d.)	262.2 (70.7)	257.5 (60.9)	254.4 (63.3)	247.8 (65.9)
Albumin, g dl^−1^, mean (s.d.)	4.4 (0.3)	4.3 (0.3)	4.3 (0.3)	4.4 (0.3)
Bilirubin, mg dl^−1^, mean (s.d.)	0.6 (0.2)	0.6 (0.3)	0.6 (0.3)	0.6 (0.3)
TC, mg dl^−1^, mean (s.d.)	186.9 (47.9)	178.1 (42.9)	181.0 (44.2)	176.8 (43.4)
HDL-C, mg dl^−1^, mean (s.d.)	45.1 (14.5)	43.8 (13.0)	43.6 (14.7)	43.2 (13.6)
LDL-C, mg dl^−1^, mean (s.d.)	115.2 (41.0)	109.1 (36.4)	111.7 (37.6)	106.8 (37.2)
apoB, mg dl^−1^, mean (s.d.)	101.1 (28.4)	95.5 (25.0)	98.1 (26.3)	95.1 (27.1)
TG, mg dl^−1^				
Mean (s.d.)	183.6 (86.2)	174.1 (93.5)	177.6 (94.4)	186.8 (119.2)
Median (Q1, Q3)	157 (126, 220)	155 (117, 206)	153 (116, 206)	158 (122, 215)
Lp(a), nmol l^−1^				
Mean (s.d.)	48.5 (73.1)	57.6 (77.6)	60.8 (77.5)	49.0 (70.2)
Median (Q1, Q3)	23 (11, 54)	21 (12, 68)	25 (11, 85)	22 (9, 46)
Baseline medications, *n* (%)				
GLP-1 RA	20 (11.7)	30 (9.3)	25 (7.6)	19 (6.0)
Metformin	71 (41.5)	138 (42.6)	137 (41.9)	136 (42.8)
Pioglitazone	3 (1.8)	3 (0.9)	3 (0.9)	1 (0.3)
SGLT2i	18 (10.5)	30 (9.3)	26 (8.0)	15 (4.7)
Statin	75 (43.9)	143 (44.1)	138 (42.2)	164 (51.6)

^a^Sex was self-reported by the patient.

^b^Includes American Indian or Alaska Native; Native Hawaiian or other Pacific Islander; and Other.

^c^Patients on thyroxine replacement therapy at baseline.

Median reported for baseline characteristics that showed high s.d.

ASCVD, atherosclerotic cardiovascular disease; FIB-4, fibrosis-4; HDL-C, high-density lipoprotein cholesterol; SGLT2i, sodium/glucose cotransporter-2 inhibitor; TC, total cholesterol.

### Primary outcome

The primary end point, incidence of TEAEs (time frame, up to 52 weeks of treatment and 4 weeks of follow-up) not being different between treatment arms was met. In total, 86.1–88.4% of resmetirom-treated patients and 81.8% of placebo-treated patients reported a TEAE during the trial (Table 2). The majority of TEAEs were mild or moderate in severity. Seventy patients experienced a serious TEAE. Approximately 20% of the serious TEAEs were related to either COVID-19 pneumonia (*n* = 8, one patient in the OL arm, two in the DB 100 mg resmetirom arm, three in the DB 80 mg resmetirom arm and two in the placebo arm) or a diagnosis code for COVID-19 (*n* = 7, 2, 1, 1 and 3 patients, respectively). No specific serious TEAEs were numerically increased in the resmetirom arms compared to placebo. TEAE rates in excess of placebo included mild or moderate diarrhea (23.5–31.2% in the resmetirom arms versus 13.8% in the placebo arm) and nausea (11.9–18.2% versus 7.9%, respectively). Diarrhea (or nausea) occurred more frequently in the resmetirom arms than the placebo arm in the first 12 weeks of treatment and the incidence was not increased in the resmetirom arms compared to placebo after 12 weeks. The median duration of diarrhea was 15–20 d in the DB resmetirom arms independent of dose. Approximately half of the diarrhea TEAEs were described as a single episode, worsening of underlying diarrhea or intermittent diarrhea. Diarrhea and nausea were further evaluated by sex and while the incidence of diarrhea was similar between sexes, nausea was more common in females than males, including in the placebo arm (Extended Data Table 2). Discontinuation from the study due to TEAEs occurred in 1.2–3.1% of patients in the resmetirom arms compared to 1.3% of patients in the placebo arm. A single patient died due to presumed cardiac arrest not related to the study drug in the 4-week follow-up period, during which, no study drug had been administered.

**Table 2:** Safety summary (safety population)

Data are *n* (%)	Resmetirom 100 mg OL (*n* = 171)	Resmetirom 100 mg DB (*n* = 324)	Resmetirom 80 mg DB (*n* = 327)	Placebo DB (*n* = 318)
≥1 TEAEs	148 (86.5)	279 (86.1)	289 (88.4)	260 (81.8)
Grade 1 (mild)	51 (29.8)	99 (30.6)	99 (30.3)	90 (28.3)
Grade 2 (moderate)	85 (49.7)	151 (46.6)	165 (50.5)	141 (44.3)
≥Grade 3 (severe)	12 (7.0)	29 (9.0)	25 (7.6)	29 (9.1)
≥1 drug-related TEAEs	63 (36.8)	119 (36.7)	114 (34.9)	77 (24.2)
≥1 serious TEAEs	7 (4.1)	24 (7.4)	19 (5.8)	20 (6.3)
≥1 drug-related serious TEAEs	0	0	0	1 (0.3)
TEAEs leading to study discontinuation	2 (1.2)	10 (3.1)	8 (2.4)	4 (1.3)
GI-related TEAEs leading to study discontinuation	0	6 (1.9)	5 (1.5)	2 (0.6)
Liver enzymes ≥3× ULN (ALT or AST)	1 (0.5)	1 (0.3)	2 (0.6)	6 (1.9)
**TEAEs occurring in** ≥**5%**				
Diarrhea	51 (29.8)	101 (31.2)	77 (23.5)	44 (13.8)
Onset ≤12 weeks	43 (25.1)	81 (25.0)	61 (18.7)	28 (8.8)
Duration, days, median (Q1, Q3)	26 (5, 64)	15 (3, 69)	20 (4, 59)	22 (3, 97)
Onset >12 weeks	8 (4.7)	20 (6.2)	16 (4.9)	16 (5.0)
Nausea	24 (14.0)	59 (18.2)	39 (11.9)	25 (7.9)
Onset ≤12 weeks	12 (7.0)	47 (14.5)	27 (8.3)	15 (4.7)
Onset >12 weeks	12 (7.0)	12 (3.7)	12 (3.7)	10 (3.1)
Abdominal pain	9 (5.3)	23 (7.1)	14 (4.3)	14 (4.4)
COVID-19	21 (12.3)	27 (8.3)	27 (8.3)	27 (8.5)
Urinary tract infection	9 (5.3)	20 (6.2)	21 (6.4)	23 (7.2)
Arthralgia	16 (9.4)	27 (8.3)	24 (7.3)	21 (6.6)
Back pain	7 (4.1)	18 (5.6)	17 (5.2)	14 (4.4)
Pain in extremity	5 (2.9)	18 (5.6)	16 (4.9)	16 (5.0)
Headache	13 (7.6)	27 (8.3)	22 (6.7)	24 (7.5)
Type 2 diabetes	8 (4.7)	21 (6.5)	18 (5.5)	14 (4.4)
Fatigue	11 (6.4)	15 (4.6)	21 (6.4)	13 (4.1)

GI, gastrointestinal.

### Secondary outcomes

**Fig. 2:**
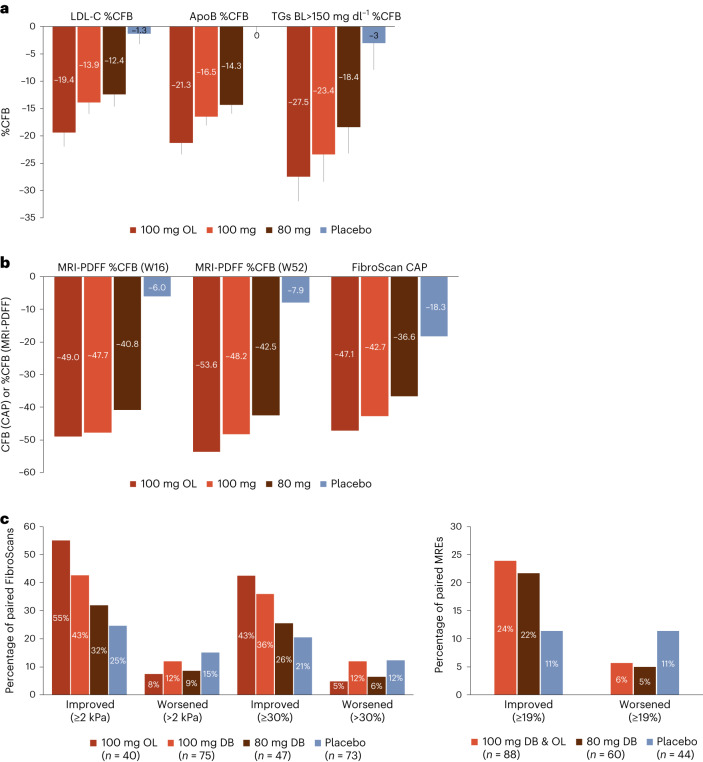
Resmetirom-mediated changes in atherogenic lipid levels and non-invasive biomarkers. **a**, Least squares mean %CFB in LDL-C, apoB and TG at week 24 (100 mg OL *n* = 169; 100 mg DB *n* = 314; 80 mg DB *n* = 320; placebo *n* = 309). TG reported in subgroup with baseline TG levels >150 mg dl^−1^ (100 mg OL *n* = 97; 100 mg DB *n* = 169; 80 mg DB *n* = 166; placebo *n* = 163). Data are presented as mean ± s.e.m. **b**, Median %CFB in hepatic fat (measured by MRI-PDFF) at weeks 16 and week 52 (100 mg OL *n* = 152; 100 mg DB *n* = 268; 80 mg DB *n* = 258; placebo *n* = 268) as well as mean CFB in FibroScan CAP at week 52 (100 mg OL *n* = 147; 100 mg DB *n* = 270; 80 mg DB *n* = 260; placebo *n* = 260). **c**, Percentage of patients whose VCTE results improved or worsened by either ≥2 kPa or ≥30% from baseline at week 52 (100 mg OL *n* = 50; 100 mg DB *n* = 102; 80 mg DB *n* = 83; placebo *n* = 107) (left). Percent of patients whose MRE results improved or worsened by ≥19% from baseline (right). mITT population. BL, baseline.

Key secondary end points were achieved for both the DB 100 mg and 80 mg resmetirom arms. At week 24, resmetirom treatment resulted in significant reductions in atherogenic lipid levels from baseline compared to placebo treatment (Fig. 2a and Table 3). At 100 mg, significant (*P* < 0.0001) reductions from baseline relative to placebo were observed in low-density lipoprotein cholesterol (LDL-C), −13.9% (2.0%) (least squares mean (s.e.m.)), apolipoprotein B; (apoB), −16.5% (1.6%) and triglycerides (TGs), −23.4% (5.0%) (baseline TG > 150 mg dl^−1^). At 80 mg, significant (*P* < 0.0001) reductions from baseline relative to placebo were observed in LDL-C, −12.4% (2.0%), apoB, −14.3% (1.6%) and TG, −18.4% (4.8%) (baseline TG > 150 mg dl^−1^). In the OL 100 mg resmetirom arm, the reductions from baseline in LDL-C, −19.4% (2.6%), apoB, −21.3% (2.1%) and TG, −27.5% (4.5%) at week 24 were numerically greater than those achieved in the DB resmetirom arms (potentially due to more missed doses of study drug in the DB arms as a result of COVID-19-related drug kit delays). Effects achieved at week 24 were maintained over 48 weeks with continued treatment (Table 3).

**Table 3:** Key secondary and secondary end points (mITT population)

	Resmetirom 100 mg OL (*n* = 169)		Resmetirom 100 mg DB (*n* = 314)			Resmetirom 80 mg DB (*n* = 320)			Placebo DB (*n* = 309)
	Least squares mean %CFB or CFB (s.e.m.)	95% CI	Least squares mean %CFB or CFB (s.e.m.)	Least squares mean difference (97.5% CI)	*P* value	Least squares mean %CFB or CFB (s.e.m.)	Least squares mean difference (97.5% CI)	*P* value	Least squares mean %CFB or CFB (s.e.m.)
**LDL-C, mg** **dl**^**−1**^									
**Week 24**	−19.4 (2.6)	−24.5 to −14.3	−13.9 (2.0)	−12.6 (−16.7 to −8.6)	<0.0001	−12.4 (2.0)	−11.1 (−15.0 to −7.2)	<0.0001	−1.3 (1.9)
Week 48	−20.5 (2.5)	−25.4 to −15.6	−13.4 (2.4)	−12.0 (−16.8 to −7.2)	<0.0001	−10.6 (2.1)	−9.2 (−14.2 to −4.3)	0.0004	−1.4 (2.4)
**LDL-C, mg** **dl**^**−1**^ **(baseline LDL-C** ≥ **100** **mg** **dl**^**−1**^**)**									
*N*	103			181			194		162
Baseline, mean (s.d.)	137.2 (35.5)			133.9 (25.6)			134.2 (28.7)		133.8 (26.3)
Week 24	−22.2 (3.0)	−28.1 to −16.3	−22.0 (2.4)	−15.9 (−20.1 to −11.7)	<0.0001	−19.0 (2.3)	−12.9 (−17.1 to −8.7)	<0.0001	−6.1 (2.4)
Week 48	−22.6 (2.9)	−28.2 to −16.9	−21.9 (3.0)	−15.6 (−20.6 to −10.7)	<0.0001	−17.3 (2.8)	−11.0 (−15.9 to −6.2)	<0.0001	−6.2 (2.8)
**ApoB, mg** **dl**^**−1**^									
**Week 24**	−21.3 (2.1)	−25.4 to −17.2	−16.5 (1.6)	−16.5 (−19.5 to −13.4)	<0.0001	−14.3 (1.6)	−14.3 (−17.4 to −11.3)	<0.0001	0.0 (1.5)
Week 48	−22.4 (2.1)	−26.4 to −18.4	−16.3 (1.8)	−15.1 (−18.7 to −11.6)	<0.0001	−13.8 (1.6)	−12.5 (−16.4 to −8.7)	<0.0001	−1.2 (1.8)
**ApoB, mg** **dl**^**−1**^ **(baseline LDL-C** ≥ **100** **mg** **dl**^**−1**^**)**									
*N*	103			181			194		162
Baseline, mean (s.d.)	115.1 (25.3)			110.4 (19.4)			112.8 (20.0)		114.2 (20.5)
Week 24	−23.6 (2.7)	−28.8 to −18.4	−22.3 (2.1)	−18.0 (−21.7 to −14.4)	<0.0001	−19.9 (2.1)	−15.6 (−19.3 to −12.0)	<0.0001	−4.2 (2.2)
Week 48	−21.7 (2.7)	−27.0 to −16.5	−23.2 (2.7)	−17.5 (−21.8 to −13.3)	<0.0001	−19.4 (2.2)	−13.8 (−18.0 to −9.6)	<0.0001	−5.6 (2.5)
**MRI-PDFF, % fat fraction**									
*n*	152			268			255		268
**Week 16**	−47.8 (3.1)	−53.8 to −41.8	−44.8 (2.6)	−38.6 (−44.6 to −32.6)	<0.0001	−41.2 (2.6)	−34.9 (−41.0 to −28.9)	<0.0001	−6.2 (2.6)
Week 52	−51.8 (3.5)	−58.6 to −45.0	−43.8 (2.9)	−33.9 (−40.7 to −27.1)	<0.0001	−38.6 (2.9)	−28.8 (−35.6 to −21.9)	<0.0001	−9.9 (2.9)
**TG, mg** **dl**^**−1**^ **(baseline TG** > **150** **mg** **dl**^**−1**^**)**									
*n*	97			169			166		163
Baseline, mean (s.d.)	232.2 (82.0)			228.3 (97.1)			236.5 (97.3)		254.0 (133.3)
**Week 24**	−27.5 (4.5)	−36.3 to −18.8	−23.4 (5.0)	−20.4 (−30.2 to −10.6)	<0.0001	−18.4 (4.8)	−15.4 (−24.9 to −5.9)	0.0015	−3.0 (4.9)
Week 48	−34.7 (4.6)	−43.8 to −25.5	−22.5 (4.5)	−22.8 (−31.0 to −14.5)	<0.0001	−22.6 (4.7)	−22.9 (−31.3 to −14.5)	<0.0001	0.3 (4.6)
**Lp(a), nmol** **l**^**−1**^ **(baseline Lp(a)** > **10** **nmol** **l**^**−1**^**)**									
*n*	130			246			244		220
Baseline, mean (s.d.)	61.1 (79.6)			72.4 (82.9)			78.0 (82.6)		64.8 (76.2)
Week 24	−28.4 (3.7)	−35.6 to −21.2	−19.7 (4.2)	−28.3 (−37.2 to −19.4)	<0.0001	−4.8 (4.1)	−13.3 (−22.3 to −4.3)	0.0037	8.5 (4.1)
Week 48	−18.7 (3.5)	−25.6 to −11.8	-33.6 (4.0)	−29.2 (−37.3 to −21.1)	<0.0001	−24.0 (4.1)	−19.6 (−27.6 to −11.6)	<0.0001	−4.4 (4.1)
**ApoCIII, mg** **dl**^**−1**^									
*n*	162			286			278		289
Baseline, mean (s.d.)	11.3 (4.8)			10.6 (4.5)			10.9 (4.5)		11.0 (4.8)
Week 24	−18.1 (3.0)	−23.9 to −12.3	−17.6 (2.5)	-18.5 (−24.3 to −12.6)	<0.0001	−11.5 (2.4)	−12.5 (−18.3 to −6.6)	<0.0001	0.9 (2.4)
Week 48	−13.6 (3.1)	−19.6 to −7.6	−14.4 (2.6)	−22.2 (−28.3 to −16.0)	<0.0001	−9.7 (2.6)	−17.5 (−23.7 to −11.2)	<0.0001	7.7 (2.6)
**FibroScan** **CAP, dBm**									
*n*	147			270			260		260
**Week 52 CFB**	−46.0 (4.8)	−55.3 to −36.6	−42.8 (4.0)	−24.4 (−33.8 to −15.1)	<0.0001	−36.7 (3.9)	−18.3 (−27.8 to −8.9)	<0.0001	−18.4 (3.9)
**FibroScan** **VCTE, kPa (baseline VCTE** ≥ **7.2** **kPa)**									
*n*	50			102			83		107
Baseline, mean (s.d.)	9.1 (2.4)			8.4 (1.6)			8.5 (1.3)		8.5 (1.8)
**Week 52 CFB**	−2.09 (0.5)	−3.0 to −1.2	−1.70 (0.4)	−0.50 (−1.3 to 0.3)	0.1710	−1.02 (0.4)	0.17 (−0.7 to 1.0)	0.6614	−1.19 (0.4)
**Liver enzymes, U** **l**^**−1**^ **(baseline ALT** ≥ **30** **IU** **l**^**−1**^**)**									
*n*	83			157			169		156
**ALT**									
Baseline, mean (s.d.)	52.5 (25.3)			51.1 (23.2)			51.9 (22.4)		55.5 (31.2)
Week 52 CFB	−14.3 (3.0)	−20.2 to −8.4	−11.9 (2.6)	−10.8 (−16.8 to −4.8)	<0.0001	−10.8 (2.5)	−9.6 (−15.7 to −3.6)	0.0004	−1.1 (2.6)
**AST**									
Baseline, mean (s.d.)	33.9 (13.9)			32.0 (12.5)			32.0 (13.2)		34.9 (17.2)
Week 52 CFB	−3.5 (2.2)	−7.9 to 0.9	−3.1 (1.9)	−4.3 (−8.7 to 0.2)	0.0341	−3.9 (1.9)	−5.0 (−9.5 to −0.4)	0.0141	1.1 (1.9)
**GGT**									
Baseline, mean (s.d.)	58.5 (66.4)			53.0 (38.1)			58.4 (50.8)		68.8 (83.0)
Week 52 CFB	−16.5 (3.9)	−24.2 to −8.7	−11.5 (3.4)	−10.0 (−17.9 to −2.0)	0.0049	−10.1 (3.3)	−8.6 (−16.5 to −0.6)	0.0157	−1.5 (3.4)
**NASH biomarkers**									
**CK-18/M30, U** **l**^**−1**^									
*n*	150			250			238		250
Baseline, mean (s.d.)	664.8 (399.4)			601.1 (357.3)			601.7 (358.4)		609.0 (410.8)
Week 52 CFB	−174.2 (31.6)	−236.2 to −112.3	−78.4 (27.1)	−82.6 (−146.8 to −18.4)	0.0040	−87.2 (26.8)	−91.4 −156.4 to −26.3)	0.0017	4.15 (26.6)
**Adiponectin, μg** **ml**^**−1**^									
*n*	126			175			173		178
Baseline, mean (s.d.)	4.9 (2.8)			4.7 (2.7)			4.7 (3.0)		4.4 (2.7)
Week 52 CFB	1.61 (0.2)	1.2 to 2.0	0.90 (0.2)	0.48 (0.02 to 0.9)	0.0195	1.12 (0.2)	0.70 (0.24 to 1.2)	0.0007	0.42 (0.2)
**Reverse T3, ng** **dl**^**−1**^									
*n*	152			265			250		257
Baseline, mean (s.d.)	18.1 (5.5)			16.3 (4.6)			17.8 (5.1)		16.8 (4.6)
Week 52 CFB	−3.34 (0.4)	−4.1 to −2.6	−3.21 (0.3)	−3.94 (−4.7 to −3.2)	<0.0001	−2.70 (0.3)	−3.43 (−4.2 to −2.7)	<0.0001	0.73 (0.3)

Lipids and lipoproteins are evaluated at week 24 and week 48 as least squares mean %CFB; other analytes as week 52 least squares mean CFB. This trial was designed to maintain an overall study-wise type I error rate of α = 0.05 for the key secondary end points only. The error rate was controlled by first splitting the overall two-sided α = 0.05 into two partitions via the Bonferroni method and then the key secondary end points tested in a prespecified hierarchical order. For the primary analysis of key secondary lipid end points, an analysis of covariance (ANCOVA) model was used to analyze each set of ten imputed datasets. The ANCOVA models for each lipid outcome included percent change from baseline to week 24 (and all other end point visits) as the dependent variable; treatment and stratification factors (presence of type 2 diabetes and ASCVD) as independent variables; and baseline lipid values as a covariable. The statistical method of estimating the treatment effect and testing for apoB and TG was similar to the LDL-C end point; a similar ANCOVA model was used whereby the baseline value of the dependent variable was included as covariate instead of LDL-C. CFB, change from baseline; %CFB, percentage change from baseline.

Additional lipid end points reported in Table 3 in DB 100 mg, reductions from baseline relative to placebo at week 24 were observed in Lp(a), −19.7% (4.2%) (least squares mean (s.e.m.)), apoCIII −17.6% (2.5) and apoB −16.5% (1.6%) and LDL-C −22.0% (2.4%) in patients with LDL-C ≥ 100 mg dl^−1^ (*P* < 0.0001 for all). At 80 mg, significant reductions from baseline relative to placebo were observed in Lp(a), −4.8% (4.1) (*P* = 0.0037), apoCIII −11.5% (2.4%) (*P* < 0.0001) and apoB −14.3% (1.6%) (*P* < 0.0001) and LDL-C −19.0% (2.3%) (*P* < 0.0001) in patients with LDL-C ≥ 100 mg dl^−1^. In the OL 100 mg resmetirom arm, the reductions from baseline were observed in Lp(a), −28.4% (3.7%), apoCIII −18.1% (3.0%) and apoB −21.3% (2.1%) and LDL-C −22.2% (3.0%) in patients with LDL-C ≥ 100 mg dl^−1^. Also, reductions were observed for multiple atherogenic species. For the DB 100 mg arms for remnant-like particle (RLP) cholesterol, −11.9% (2.6%), very low-density lipoprotein (VLDL) cholesterol, −12.9% (5.3%) and atherogenic lipoprotein particles LDL, −16.7% (1.7%) and small LDL particles, −17.1% (2.9%) were reduced compared to the placebo arm (all *P* < 0.0001). Data for 80 mg DB and OL are also reported (Extended Data Table 3 and Supplementary Table 3).

As a key secondary end point, at week 16 resmetirom treatment showed a relative reduction in hepatic fat compared to placebo treatment (least squares mean percent change from baseline (95% confidence interval (CI)) for OL 100 mg, −47.8% (−53.8% to −41.8%); DB 100 mg, −45.1% (−50.3% to −39.9%); 80 mg, −41.4% (−46.6% to −36.2%); placebo, −6.5% (−11.7% to −1.3%); *P* < 0.0001 versus placebo for all three comparisons (Fig. 2b and Table 3). The significant relative reduction in hepatic fat achieved by week 16 was sustained over 52 weeks with continued resmetirom treatment (Fig. 2b and Table 3). As an additional secondary end point, at week 52 the least squares mean relative reduction from baseline (95% CI) in hepatic fat was −51.8% (−58.6% to −45.0%) in the OL 100 mg resmetirom arm. Similarly, as a key secondary end point, at week 52 hepatic fat as estimated by continuous attenuation parameter (CAP) was reduced (least squares mean percent change from baseline (95% CI) for OL 100 mg −46.0% (−55.3% to −36.6%); DB 100 mg −42.8% (−33.8% to −15.1%); 80 mg −36.7% (−27.8% to −8.9%); *P* < 0.0001 versus placebo for three comparisons (Fig. 2b and Table 3).

**Extended Data Fig. 1:**
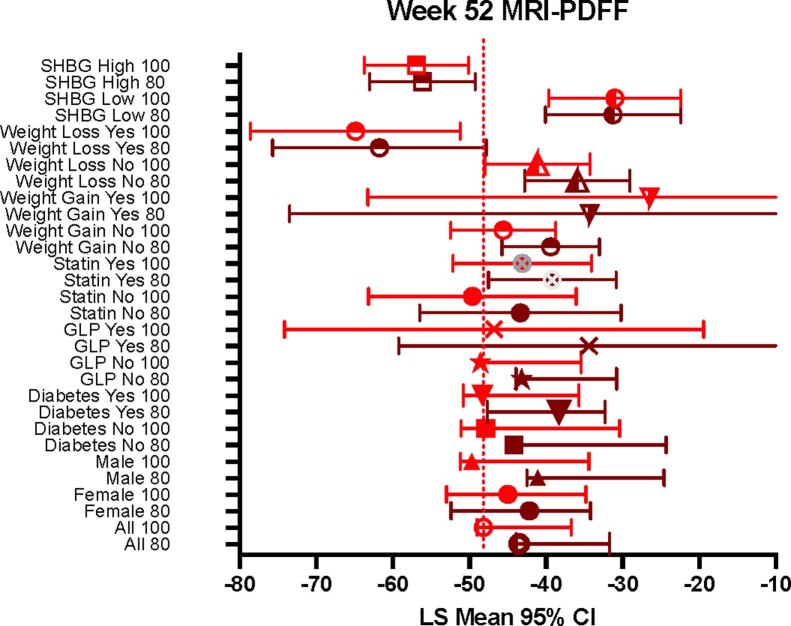
Resmetirom-mediated reduction in hepatic fat. Least squares (LS) mean change from baseline (95% confidence interval (CI)) in hepatic fat (measured by magnetic resonance imaging-proton density fat fraction (MRI-PDFF)) at Week 52 in patient subgroups of the double-blind 100 mg resmetirom and double-blind 80 mg resmetirom arms. Marker shows median. High SHBG, population with ≥120% increase from baseline in SHBG.

MRI-PDFF subgroup analyses demonstrated that resmetirom treatment reduced hepatic fat from baseline at week 52 in all key patient subgroups (Extended Data Fig. 1). In particular, weight loss ≥5% in combination with resmetirom treatment or high exposure to resmetirom measured by the sex hormone-binding globulin (SHBG) response as described previously^14^ (defined in this study as ≥120% increase from baseline in SHBG, which corresponds to the upper two tertiles of the SHBG response observed with 100 mg resmetirom) was associated with greater reduction in hepatic fat. In contrast, weight gain ≥5% or lower exposure to resmetirom (defined as <120% increase in SHBG) showed lower hepatic fat reduction with resmetirom. In general, 80 mg resmetirom was less effective than 100 mg resmetirom at reducing hepatic fat; however, the effect of 80 mg was similar to 100 mg in females and in patients with ≥120% increase in SHBG.

In this trial, approximately one-third of randomized patients had a baseline liver stiffness measurement (LSM) via vibration-controlled transient elastography (VCTE) that met prespecified criteria for analysis as a key secondary end point (≥7.2 kPa (which has been shown to have a ≥ 90% positive predictive value for moderate fibrosis (F2)) (51 patients in the OL arm, 102 in the DB 100 mg resmetirom arm, 83 in the DB 80 mg resmetirom arm and 107 in the placebo arm). Although directionally showing a treatment effect in the DB 100 mg resmetirom arm, the mean change from baseline in VCTE was not significantly different between the resmetirom and placebo arms at week 52 (Table 3). A responder analysis was subsequently conducted to reduce the influence of measurement variability. This analysis showed that a numerically greater percentage of patients in the resmetirom arms achieved either a ≥2 kPa reduction from baseline (32–55% in the resmetirom arms versus 25% in the placebo arm) or a ≥30% reduction from baseline (26–43% versus 21%, respectively) in VCTE at week 52 (Fig. 2c). A similar responder analysis was conducted for liver stiffness measured by magnetic resonance elastography (MRE). The percentage of patients who achieved a ≥19% reduction from baseline in MRE at week 52 was greater in the resmetirom arms compared to the placebo arm (22–25% versus 11%) and numerically fewer resmetirom-treated patients had a ≥19% increase in MRE (Fig. 2c).

**Fig. 3:**
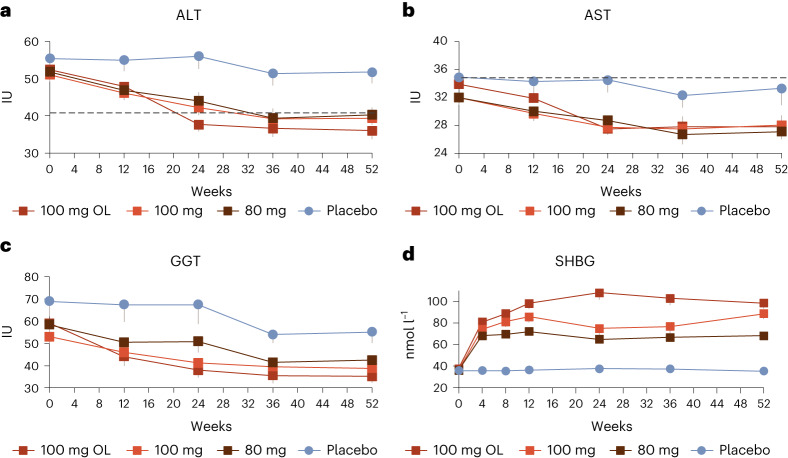
Resmetirom-mediated changes in markers of liver injury and sex-hormone bindingglobulin. **a**–**d**, Mean ALT (**a**), AST (**b**) and GGT (**c**) in the subgroup with baseline ALT levels ≥30 IU l^−1^ and SHBG (nmol l^−1^) (**d**) levels over time. Shown are observed data (100 mg OL *n* = 83; 100 mg DB *n* = 157; 80 mg DB *n* = 169; placebo *n* = 156). Data are presented as mean ± s.e.m.

Markers of liver injury, additional secondary end points in the trial, were improved with resmetirom treatment. Mean baseline liver enzymes were low in this population (mean baseline alanine aminotransferase (ALT), ~37 U l^−1^ (upper limit of normal (ULN) 41 U l^−1^)). In the subgroup of patients with ALT ≥ 30 IU l^−1^ at baseline, ALT, aspartate aminotransferase (AST) and γ-glutamyl transferase (GGT) levels were significantly reduced from baseline in the OL 100 mg and DB 100 mg and 80 mg resmetirom arms compared to the placebo arm at week 52 (*P* < 0.05 versus placebo for all) (Table 3). Figure 3 shows mean ALT, AST and GGT levels over the 52-week treatment period in patients with baseline ALT ≥ 30 IU l^−1^. SHBG increases reflect the degree of THR-β activation in the liver and correlate with resmetirom exposure. A time course of the level of SHBG demonstrates that SHBG gradually increased with a plateau at week 12 that seemed to correlate with a plateau in ALT/AST reduction at week 24. Lower levels of SHBG were apparent in the DB arm as compared to the OL 100-mg arm, consistent with the COVID-19-related drug kit delays that occurred in the DB arms.

At week 52, biomarkers of hepatocyte injury were significantly improved from baseline among resmetirom-treated versus placebo-treated patients (Table 3). Cytokeratin-18 (CK-18) fragments were markedly reduced from baseline with resmetirom treatment (−78.4 to −87.2 U l^−1^) compared to an increase from baseline with placebo (4.15 U l^−1^; *P* < 0.01 versus placebo at both doses). Adiponectin levels were significantly increased from baseline among resmetirom-treated patients relative to placebo-treated patients (0.90–1.12 versus 0.42 μg ml^−1^, respectively; *P* < 0.05 versus placebo at both doses). Levels of reverse triiodothyronine (rT3) were reduced from baseline in the resmetirom arms and increased in the placebo arm, resulting in a significant treatment difference at week 52 (*P* < 0.0001 versus placebo for both doses) (Table 3). The enhanced liver fibrosis (ELF) score was evaluated in a subset of patients with a baseline score of >9.8 (Extended Data Table 4). No significant difference relative to placebo was observed for the total score but a reduction in tissue metalloproteinase-1 (TIMP-1) was observed. PROs were assessed utilizing the chronic liver disease health questionnaire. There were no observed differences between the resmetirom and placebo groups (data not shown).

### Exploratory outcome

ALT increases of ≥3 × ULN occurred in fewer patients in the resmetirom arms compared to the placebo arm (0.5% (OL 100 mg resmetirom), 0.3% (DB 100 mg resmetirom) and 0.6% (DB 80 mg resmetirom) versus 1.9% (placebo)). As a marker of potential efficacy, liver enzymes were reduced from baseline over time in the resmetirom arms compared to placebo. Safety observations related to potential thyroid axis or thyroid hormone effects showed no increase in signs or symptoms of hypothyroidism or hyperthyroidism relative to placebo. At week 52, minor reductions in body weight from baseline were noted in all treatment arms with no significant difference between the DB resmetirom and placebo arms (Extended Data Table 5). No adverse cardiovascular (CV) or bone TEAEs (fracture) were noted with resmetirom. Blood pressure, including systolic and diastolic, generally decreased from baseline by 2–3 mm Hg in the resmetirom arms (Extended Data Table 5). No change in heart rate (based on electrocardiogram (ECG)) was observed in resmetirom-treated patients compared to placebo-treated patients and no arrythmias were noted.

Minimal reductions from baseline in prohormone FT4 levels were observed in the resmetirom arms. No effects on active hormone free triiodothyronine (FT3) or thyroid-stimulating hormone (TSH) were noted in the resmetirom arms. Sex hormone levels after 52 weeks of resmetirom treatment are shown in Extended Data Table 6 and Supplementary Table 3. Thyroid hormone levels after 52 weeks of resmetirom treatment are reported in Extended Data Table 6 and Supplementary Table 4.

**Extended Data Fig. 2:**
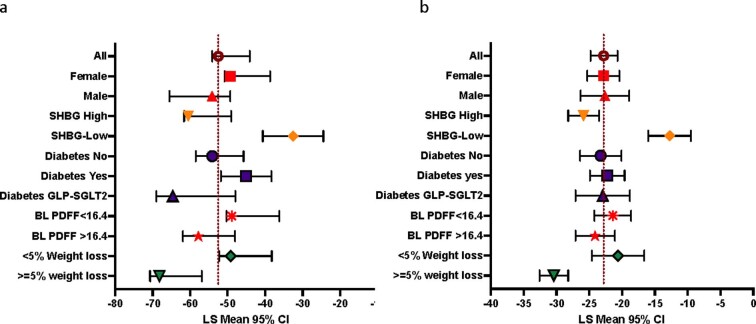
Resmetirom-mediated reduction in proton density fat fraction and liver volume. Least squares (LS) mean change from baseline (95% confidence interval (CI)) in PDFF (a) and liver volume (b) at Week 52 in patient subgroups of the open-label 100 mg resmetirom arm. Marker shows median. High SHBG, population (~two-thirds) with ≥120% increase from baseline in SHBG.

Liver volume was evaluated in the OL 100 mg resmetirom arm at week 52. Liver volume was reduced from the baseline by a mean of 21% and 23% following 16 and 52 weeks of resmetirom treatment, respectively (Extended Data Fig. 2). Furthermore, reductions in liver volume were observed in all key patient subgroups of the OL arm. Correcting hepatic fat reduction for reduced liver volume led to an average hepatic fat reduction of 61% in the OL arm.

## Discussion

There are currently no approved pharmacological therapies for NASH. Rather, management focuses on lifestyle modification, including diet and exercise and treatment of comorbidities such as obesity, type 2 diabetes and dyslipidemia. In addition, NASH is a chronic condition which may require lifelong therapy to slow or prevent progression of the disease. The blinded, placebo-controlled data from the 52-week MAESTRO-NAFLD-1 trial reinforce the safety data reported from the 36-week resmetirom phase 2 NASH trial but in a larger population over a longer treatment period.

Resmetirom was well tolerated at both 80 and 100 mg once-daily doses over 52 weeks of treatment in this trial. There were no increases in serious TEAEs or notable imbalances in specific serious TEAEs in the resmetirom arms compared to placebo. Consistent with previous data, excess TEAEs were mostly gastrointestinal in nature (diarrhea and nausea) and characterized as mild or moderate at the initiation of resmetirom treatment^14^. Diarrhea was of short duration (approximately 2 weeks) and the incidence of this TEAE was not increased relative to placebo after week 12. Few patients discontinued the study due to these TEAEs. The incidence of nausea was more frequent in females relative to placebo (with females in the placebo arm being more frequent than males in the placebo arm).

In patients with NASH, THR-β signaling within the liver is diminished, impacting lipid metabolism, fatty acid oxidation and energy production, potentially resulting in worsening NASH and liver fibrosis^13^. The lipotoxicity that occurs in NASH induces intrahepatic hypothyroidism resulting in reduced conversion of prohormone T4 to active hormone T3 in favor of increased conversion of T4 to the inactive metabolite rT3 (refs. ^13,15^). Resmetirom is a liver-targeted THR-β-selective agonist designed to address this underlying pathophysiology in patients with NASH. The minimal but significant reduction in FT4 has been previously noted and is thought to be due to increased conversion of T4 to T3 and reduction in rT3 in the liver that is mediated by *DIO1* (a known target gene of THR-β) (refs. ^13,14^). Thyroxine treatment does not improve this deficiency; in fact, patients with NASH treated with thyroxine (T4) have more exaggerated elevations of rT3 and reductions in the FT3/rT3 ratio^15^. As expected, in MAESTRO-NAFLD-1 resmetirom treatment significantly reduced rT3 levels from baseline by week 52 in both thyroxine-treated and euthyroid patients compared to placebo treatment and improved the FT3/rT3 ratio, both indicative of normalization of thyroid hormone function in the liver.

While resmetirom was specifically designed to target the liver to correct the dysfunctional THR-β signaling in patients with NASH, it is critical to ensure that resmetirom does not affect the more widely expressed THR-α receptor, which is responsible for thyroid hormone activity in the heart and bone. No signs or symptoms related to systemic hyperthyroidism or hypothyroidism, or central thyroid axis changes were noted. As shown previously, no change in heart rate was observed^14^. Rather than increases in blood pressure, as would be expected with excess THR-α activity, small reductions in both systolic and diastolic blood pressure were observed.

CV disease is the most common cause of death in patients with NASH, thus the CV effect of potential NASH therapies is to be considered^16^. Elevated atherogenic lipids and lipoproteins have been shown to contribute to increased CV risk^17^. In this trial, reductions in LDL-C, apoB and TG levels were achieved with both DB 80 and 100 mg resmetirom; similar effects were observed with OL 100 mg resmetirom. Resmetirom significantly reduced atherogenic lipids/lipoproteins from baseline, including apoCIII, Lp(a), RLP and VLDL cholesterol when compared to placebo treatment. Improvements in the lipid/lipoprotein profile were maintained throughout the 52-week treatment period.

Resmetirom reduced hepatic fat from baseline compared to placebo when assessed by either MRI-PDFF or CAP. The 100-mg resmetirom dose showed consistently greater reductions in hepatic fat (measured by MRI-PDFF) than 80 mg; however, patients who achieved a target level of SHBG increase (≥120% increase from baseline) on 80 or 100 mg resmetirom showed a similar reduction in hepatic fat. In the previous phase 2 trial, an MRI-PDFF reduction of ≥30% or ≥50% by resmetirom was shown to be significantly related to the magnitude of NASH reduction, including all components of NASH and fibrosis reduction^12,14^. Previously, it was shown that similar PDFF reductions were achieved with resmetirom independent of the fibrosis stage^14^. Furthermore, the reduction in liver volume observed with resmetirom in this trial suggests even greater efflux of fat from the liver than that estimated by hepatic fat before correction for liver volume.

The limited number of patients who had a baseline FibroScan VCTE LSM that met the criteria for analysis precluded the ability to evaluate the effect of resmetirom on fibrosis in this patient population; however, responder analyses suggested fibrosis was improved from baseline by week 52 in the resmetirom arms compared to the placebo arm (based on reduction in either FibroScan VCTE LSM or MRE). In support of a potential improvement in fibrosis, multiple liver injury biomarkers, such as TIMP-1 (a component of the ELF score), CK-18 and adiponectin, were significantly improved compared to placebo after 52 weeks of resmetirom treatment.

Limitations of the MAESTRO-NAFLD-1 trial include the impact of COVID-19-related dose interruptions on the evaluation of safety and efficacy in the DB arms. Another limitation was the early fibrosis stage of patients, which restricted evaluation of the effect of resmetirom on noninvasive measures of fibrosis, particularly VCTE and ELF score and the inability for noninvasive testing to definitively stage the disease in these patients; however, from the perspective of safety (primary end point of the study), MAESTRO-NAFLD-1 evaluated a high-risk population with significant comorbidities (almost half with type 2 diabetes; the majority with hypertension, obesity and high CV risk).

In conclusion, resmetirom did not produce a difference in TEAEs relative to placebo over 52 weeks of treatment. The positive results from MAESTRO-NAFLD-1 are supportive of resmetirom’s safety and tolerability profile in patients with NAFLD.

## Methods

### Study design and participants

**Extended Data Fig. 3:**
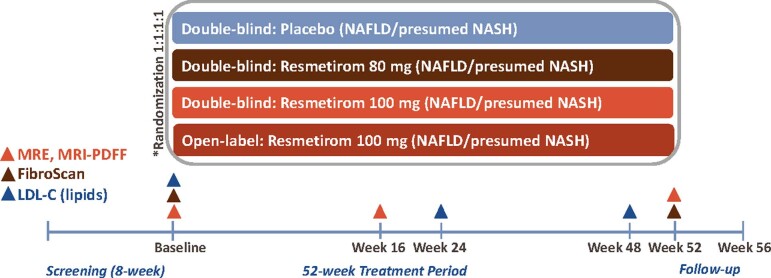
Study Design. *Randomization was stratified by type 2 diabetes status and by history of documented atherosclerotic cardiovascular disease. The first 30 patients enrolled in the MAESTRO-NAFLD-1 trial were enrolled in the open-label 100 mg resmetirom (including any patients taking thyroxine >75 mcg). After the first 30 patients were enrolled in the open-label 100 mg resmetirom arm, subsequent patients were randomized 1:1:1:1 to 3 double-blind arms (100 mg resmetirom, 80 mg resmetirom, or placebo) or the open-label 100 mg resmetirom arm. Randomization to the open-label arm was discontinued when the target number of patients was achieved (July 1, 2020). Thereafter, eligible patients were randomized 1:1:1 to the 3 double-blind arms (100 mg resmetirom, 80 mg resmetirom, or placebo).

MAESTRO-NAFLD-1 (ClinicalTrials.gov identifier NCT04197479) was a randomized, DB, placebo-controlled phase 3 trial evaluating the safety and tolerability of resmetirom in patients with NAFLD (presumed NASH). The trial consisted of a screening period of up to 8 weeks, a 52-week treatment period and a 4-week follow-up period (Extended Data Fig. 3). Visits were conducted every 4 weeks. In addition to the three DB arms (100 mg resmetirom, 80 mg resmetirom and placebo), MAESTRO-NAFLD-1 included three OL arms in patients with (1) noncirrhotic NASH; (2) well-compensated NASH cirrhosis (to be reported separately); and (3) moderate renal impairment (to be reported separately).

Male and female adults ≥18 years of age with ≥3 metabolic risk factors could screen for inclusion in MAESTRO-NAFLD-1. At sites participating in MAESTRO-NASH, patients who failed the screen for MAESTRO-NASH but had confirmed NAFLD (earlier stage or F2/F3 with NAFLD activity score (NAS) < 4) could enroll in MAESTRO-NAFLD and patients with FibroScan VCTE LSM ≥ 5.5 and <8.5 kPa and FibroScan CAP ≥ 280 dB m^−1^ without liver biopsy could screen for inclusion in MAESTRO-NAFLD-1. At sites not participating in MAESTRO-NASH, FibroScan VCTE/LSM ≥ 5.5 kPa and FibroScan CAP ≥ 280 dB m^−1^ were required. Acceptable standard blood chemistry and hematology screening laboratory results and the presence of ≥8% hepatic fat (measured by MRI-PDFF) were screening requirements. Initially, thyroxine treatment was limited to ≤75 μg d^−1^ in the DB arms (except in patients post-thyroidectomy), whereas all doses of thyroxine treatment were permitted in the OL 100 mg resmetirom arm. Patients were excluded who had a history of significant alcohol consumption for ≥3 months within 1 year of screening, history of bariatric surgery or intestinal bypass surgery in the 5 years before randomization, experienced a ≥5% weight gain or loss within 12 weeks of randomization, HbA1c > 9.0%, diagnosis of hepatocellular carcinoma, model for end-stage liver disease score ≥12, ALT > 250 U l^−1^ or receiving treatment with pioglitazone >15 mg d^−1^. Pioglitazone was permitted if the dose was ≤15 mg d^−1^ and stable for ≥12 weeks before randomization. Treatment with GLP-1 RAs was permitted if the dose was stable for ≥24 weeks before screening. High-dose vitamin E treatment (>400 IU d^−1^) was permitted if the dose was stable for ≥24 weeks before randomization.

### Inclusion criteria (DB and OL noncirrhotic arms)

We only evaluated patients for study participation if they met the prescreening criteria. Patients who did not initially meet the eligibility criteria may be retested, based on investigator judgment, to determine whether they qualify to participate. Patients who met all of the following criteria were eligible to participate in the study:

1. Must be willing to participate in the study and provide written informed consent.
2. Male and female adults ≥18 years of age.
3. Female patients are eligible if they are of reproductive potential and have a negative serum pregnancy test (β-human chorionic gonadotropin), are not breastfeeding and do not plan to become pregnant during the study and agree to use two highly effective birth-control methods during the study OR if they are not of childbearing potential (surgically (bilateral oophorectomy, hysterectomy or tubal ligation) or are naturally sterile (>12 consecutive months without menses)). Highly effective birth-control methods include condoms with spermicide, diaphragm with spermicide, hormonal and non-hormonal intrauterine device, hormonal contraception (estrogens stable ≥3 months), a vasectomized male partner or sexual abstinence (defined as refraining from heterosexual intercourse, from the time of screening throughout the study and for at least 30 d after the last dose of study drug administration). Reliance on abstinence from heterosexual intercourse is acceptable only if it is the patient’s habitual practice.
4. Male patients who are sexually active with a partner of childbearing potential must either be sterile (vasectomy with history of a negative sperm count at least 90 d following the procedure); practice total abstinence from sexual intercourse as the preferred lifestyle (periodic abstinence is not acceptable); use a male condom with any sexual activity; or agree to use a birth-control method considered to be appropriate by the investigator (such as one of the methods identified above for female patients of childbearing potential) from the time of screening until 30 d after the last dose of study drug administration. Male patients must agree not to donate sperm for a period of 30 d after the last dose of study drug administration.
5. For suspected or confirmed diagnosis of NASH/NAFLD suggested by the historical data, they must meet one of the following criteria:
  FibroScan with kPa ≥ 5.5 and <8.5; CAP ≥ 280 dB m^−1^ OR MRE ≥ 2.0 and <4.0; MRI-PDFF ≥ 8% liver fat consistent with steatosis and fibrosis stage ≥1 and <4.
  Recent liver biopsy (within past 2 years) documenting NASH/NAFLD with steatosis showing one of the following:
    NAS ≥ 4, steatosis ≥ 1, with fibrosis stage 0 (F0) OR with F1A/1C and PRO-C3 < 14.
    NAS < 4, steatosis ≥ 1, with fibrosis stage ≤3.
    NAS ≥ 4, steatosis ≥ 1, fibrosis stage ≤3 without ballooning.
      Note: following the completion of enrollment of the DB arms, patients meeting all other criteria who have a liver biopsy result from MGL-3196-11 with the following may be enrolled in the OL active treatment arm of MGL-3196-14 (100 mg dose):
    NAS = 3, steatosis 1, ballooning 1, inflammation 1 with F2 or F3.
    NAS = 3, ballooning 0 with F2 or F3.
  Note: if patient is F0, they must also, within the last 6 months, have a FibroScan with kPa ≥ 5.5 OR MRE ≥ 2.0 (no upper limit).
  Note: in patients on thyroxine >75 μg, a FibroScan with kPa ≥ 5.5 is eligible (no upper limit).
  Note: in patients with glomerular filtration rate (GFR) ≥ 30 and <45 (OL treatment arm). a FibroScan with kPa ≥ 5.5 is eligible (no upper limit).
  Note: eligibility based on meeting inclusion criterion 5 should be determined based on historic medical and laboratory (FibroScan, liver biopsy) data and should be determined before informed consent is provided and the screening visit.
  Note: FibroScan does not need to be repeated at screening if conducted at prescreening and/or a historical FibroScan was conducted in the previous 3 months (including study MGL-3196-11). A historic liver biopsy must not be accompanied by a significant decrease in body weight ≥5% since the biopsy or treatment with a new concomitant medication that might affect steatosis.
6. Patients must meet inclusion criterion 0 before obtaining an MRI-PDFF (unless a patient has an existing qualifying MRI-PDFF). MRI-PDFF fat fraction ≥8% obtained during the screening period (baseline MRI-PDFF) or a historic MRI-PDFF ≤ 8 weeks (+3 d) old at the time of randomization.
  Note: an eligible MRI-PDFF with fat fraction ≥8% must be obtained for the baseline value (does not need to be repeated if conducted in the ≤8 weeks (+3 d) before anticipated randomization and there has not been >5% weight change in that interval). Patients with contraindications to an MRI-PDFF (for example, metal prosthetics, uncontrolled documented claustrophobia or where body weight exceeds the limit of the machine) examination or who are screened at an investigative site where MRI-PDFF is not available are eligible for this study if they have a FibroScan with CAP ≥ 300 dB m^−1^ or a CAP ≥ 280 with a historic liver biopsy showing steatosis ≥1.
  Note: if a historic liver biopsy shows steatosis of 0 and there has been no recent change in metabolic status such as weight gain, the patient is not eligible to participate unless the current MRI-PDFF is ≥8%.
7. Must be on stable, standard care dyslipidemia therapy for ≥30 d before randomization that can be maintained for the entire study except for noted exceptions. Statins should be taken in the evening for at least 2 weeks before randomization and continue to be taken in the evening for the duration of the study. Patients recommended for dyslipidemia therapy but who are intolerant of statins or other dyslipidemia therapy are allowed. Permitted dyslipidemia therapy includes:
  Rosuvastatin ≤20 mg daily.
  Atorvastatin ≤40 mg daily.
  Simvastatin ≤20 mg daily.
  Pravastatin ≤40 mg daily.
  Lovastatin ≤40 mg daily.
  Pitavastatin ≤2 mg daily.
  Ezetimibe alone or in combination with any of the above statins.
  Omega III fatty acids.
  Bile acid sequestrants (for example, cholestyramine (Questran or Prevalite), colestipol (Colestid or Flavored Colestid) and colesevelam (Welchol)) are permitted if taken at least 4 h after or 4 h before the dose of study drug.
  The fenofibrate dose must be stable for at least 6 weeks before randomization and is allowed for patients with a history of and/or ongoing very high TGs (>500 mg dl^−1^) based on investigator discretion. Unless taking fenofibrate for a history of and/or ongoing very high TGs (500 mg dl^−1^), fenofibrate is excluded throughout the study. For patients on fenofibrate in which previous high TG values >500 mg dl^−1^ cannot be located, the sponsor, in consultation with the investigator may review the case details to evaluate the justification for the fenofibrate indication on a case-by-case basis.
  Note: because lipid end points are assessed during the study, lipid therapies must be stable for the duration of the 52-week study. Lipids are blinded during the study and monitoring lipid levels at local laboratories outside the clinical trial is discouraged. Safety reasons that may result in an increase/modification in lipid therapy during the study include the occurrence of a laboratory alert for TGs > 1,000 or another acute indication for modifying lipid therapy such as a CV event. Statins and other lipid therapy may be reduced, switched to an alternative statin or cholesterol-lowering medication or discontinued secondary to AEs during the study.
  Note: patients already enrolled who are taking fenofibrate even if not for very high TGs may remain in the study, because there are no safety concerns in most patients taking fenofibrate.
  Note: other stable dyslipidemia therapies not specifically listed, such as PCSK9 inhibitors, are allowed.
  Note: patients with dyslipidemia therapy that was initiated <30 d before expected randomization may be rescreened after the dyslipidemia therapy is stabilized. In the case that a lipid-lowering medication is discontinued before randomization, the patient would be able to randomize 1 week after the discontinuation.
8. Estimated GFR ≥ 45 (DB treatment arms) or ≥30 and <45 (OL treatment arm) by the modification of diet in renal disease 6-variable (MDRD-6) formula.

### Exclusion criteria

Patients who meet any of the following criteria will be excluded from participation in the study. Patients who do not initially meet eligibility criteria may be retested or rescreened, based on investigator judgment, to determine whether they qualify to participate.

1. History of significant alcohol consumption for a period of more than three consecutive months within 1 year before screening. Note: significant alcohol consumption is defined as equal to or greater than approximately two alcoholic drinks per day for males and approximately 1.5 alcoholic drinks per day for females. One alcoholic drink is equal to 12 ounces (355 ml) of 5% alcohol by volume (ABV) beer, 5 ounces (148 ml) of 12% ABV wine or 1.5 ounces (44.4 ml) of 40% ABV distilled spirits. Note: Cabohydrate-deficient transferrin (CDT) will be measured at screening and if elevated by ≥3.0%, patients will be excluded unless another reason for CDT elevation is provided. If CDT is elevated >2.47 and <3.0, additional patient history should be obtained to rule out alcohol consumption inconsistent with the protocol exclusion limit. Significant alcohol consumption is not allowed for the duration of the study.
2. Regular use of drugs historically associated with NAFLD, which include, but are not limited, to the following: amiodarone, methotrexate, systemic glucocorticoids at greater than 5 mg d^−1^, tamoxifen, estrogens at doses greater than those used for hormone replacement or contraception, anabolic steroids except testosterone replacement, valproic acid and known hepatotoxins for more than 4 weeks within the last 8 weeks before the initial screening.
3. Thyroid diseases:
  - Active hyperthyroidism. Note: patients with a history of hyperthyroidism are eligible to participate.
  - Untreated clinical hypothyroidism defined by TSH > 7 IU l^−1^ with symptoms of hypothyroidism or >10 IU l^−1^ without symptoms. Note: TSH may be repeated once, and if >10 IU l^−1^, even with normal FT4 levels, patients may be stabilized on thyroxine replacement therapy and rescreened for eligibility. Patients with TSH > 7 and <10 with no symptoms of hypothyroidism are eligible and TSH may be monitored normally.
  - Subclinical hypothyroidism and patients on stable thyroxine therapy are eligible to participate. During screening and after randomization, small adjustments of the thyroxine dose (12.5–25 μg every 4 weeks) are allowed as per the usual care.
  Note: investigators are asked to review TSH levels, particularly in OL and DB patients on thyroxine, at screening, baseline and throughout the study to determine whether to make dose adjustments in thyroxine (or recommend thyroxine dose adjustments to the patient’s primary care physician (PCP)). Small dose adjustments in thyroxine (12.5 to 25 μg every 4 weeks) are expected and are consistent with ‘stable’ thyroxine therapy. Patients with Hashimoto’s thyroiditis may have residual thyroid function and typically require a dose that is lower than patients with a complete thyroidectomy. Target TSH level may be >1 in patients at high CV risk or established CV disease.
  Note: NASH/NAFLD is associated with reduced conversion of prohormone T4 to the active hormone T3 in the liver and decreased plasma free T3/reverse T3 ratio. Most patients on thyroxine enrolled in the study will not require a reduction in thyroxine dose. Some patients with NAFLD may require a small reduction in thyroxine dose during screening or after randomization to maintain TSH at target due at least in part to more efficient conversion of T4 to T3 in the liver (and reduced reverse T3) that occurs with resmetirom treatment and/or increased diet/exercise.
4. History of bariatric surgery or intestinal bypass surgery within the 5 years before randomization or planned during the conduct of the study.
5. Weight gain or loss ≥5% total body weight within 12 weeks before randomization. Note: this includes the screening period.
6. HbA1c > 9.0%. Note: patients with HbA1c > 8.0% and ≤10.0% should have documented efforts to control HbA1c to ≤8.0%. If there is no previous documentation of efforts to control HbA1c, patients may be treated with new or higher doses of existing diabetic medication(s) and are eligible for rescreening 6 weeks after initiating a new antidiabetic therapy if the repeated HbA1c is ≤9.0%. Patients must be on stable treatment for all diabetes medications, including any new doses or medications, for ≥30 d before randomization.
  Note: stable diabetes therapy is expected at the time of randomization, but may be modified after randomization, including increases in dose, switching or adding an additional antidiabetic if medically indicated (with the exception of adding GLP-1 agonists) to maintain good HbA1c control during the study. Investigators should enforce diet and exercise as described in the protocol.
  Note: insulin doses may be altered by up to 10% during the screening period.
7. GLP-1 agonist therapy (for example, exenatide, liraglutide, lixisenatide, albiglutide, dulaglutide, semaglutide and albiglutide) unless stable dose for 24 weeks before screening. GLP-1 therapeutics may not be initiated, or doses increased during the study. A switch within class from a GLP-1 agonist to a different GLP-1 agonist at a dose assessed as equivalent would be allowed during the study.
8. Use of high-dose vitamin E (>400 IU d^−1^) unless stable for ≥24 weeks before randomization. Vitamin E can be discontinued or decreased but dose cannot be increased during the study.
9. Presence of cirrhosis on liver biopsy defined as stage 4 fibrosis is excluded from randomized arms.
10. Diagnosis of hepatocellular carcinoma.
11. Model for end-stage liver disease score ≥12 due to liver disease. Note: score of ≥12 must be the result of liver disease to be exclusionary, not isolated laboratory abnormalities such as elevated creatinine due to chronic kidney disease, international normalized ratio (INR) abnormality secondary to anticoagulants or laboratory error and bilirubin elevation due to Gilbert’s syndrome.
12. Hepatic decompensation or impairment defined as presence of any of the following:
  - History of esophageal varices, ascites or hepatic encephalopathy.
  - Serum albumin <3.5 g dl^−1^, except as explained by nonhepatic causes.
  - INR > 1.4 unless due to therapeutic anticoagulants or laboratory error. Note: INR may be repeated once to reassess eligibility.
  - Total bilirubin > 1.5 × ULN = 1.2 mg dl^−1^. Note: patients with Gilbert syndrome are eligible with a total bilirubin above 1.5 × ULN if reticulocyte count is within normal limits (typically 0.5–2.5%), hemoglobin is within normal limits unless due to chronic anemia and unrelated to hemolysis (typically 13.5–17.5 g dl^−1^ for men; 12.0–15.5 g dl^−1^ for women) and direct bilirubin is <20% of total bilirubin.
  Chronic liver diseases:
  - Primary biliary cholangitis.
  - Primary sclerosing cholangitis.
  - Hepatitis B positive: as defined by the subject testing positive for Hepatitis B surface antigen, core antibody and positive for hepatitis B virus infection. A patient could also be excluded if hepatitis B DNA is detected.
  - Hepatitis C as defined by presence of hepatitis C virus (HCV), HCV antibody and positive HCV RNA (tested for known cured HCV infection or positive HCV antibody at screening). Note: patients who are HCV antibody positive and HCV RNA negative who have a history of clearly documented HCV infection (history of positive HCV RNA) are eligible to participate if previous treatment for HCV was given and they have a documented sustained virologic response of at least 2 years before anticipated randomization.
  - History or evidence of current active autoimmune hepatitis.
  - History or evidence of Wilson’s disease.
  - History or evidence of α-1-antitrypsin deficiency.
  - History or evidence of genetic hemochromatosis (hereditary or primary).
  - Evidence of drug-induced liver disease, as defined on the basis of typical exposure and history.
  - Known bile duct obstruction.
  - Suspected or proven liver cancer.
13. Has an active autoimmune disease, including actively treated lupus, rheumatoid arthritis, inflammatory bowel disease or autoimmune hepatitis, requiring systemic treatment within the past 12 weeks or a documented history of clinically severe autoimmune disease, including autoimmune liver disease or a syndrome that requires systemic steroids or immunosuppressive agents. Note: patients with vitiligo or resolved childhood asthma/atopy would be an exception to this rule. Patients who require intermittent use of bronchodilators, topical, inhaled or intranasal corticosteroids, or local steroid injections are not excluded from the study.
  Note: as evaluation of safety, not efficacy, is the major objective being evaluated in patients with moderate renal impairment (eGFR ≥ 30 and <45) enrolled in the OL arm, patients with autoimmune diseases such as rheumatoid arthritis who are on systemic therapies may be eligible on a case-by-case basis as long as the systemic therapy for the autoimmune disease is not specifically excluded.
14. Serum ALT > 250 U l^−1^.
  Note: given the intrinsic variability in ALT and AST in patients with NASH, investigators should use the following guide in an attempt to establish a relatively stable baseline for ALT and AST. Investigator discretion is allowed. Documented historical (3 weeks to 6 months before study entry) ALT and AST levels consistent with the screening ALT and AST values may help establish a stable baseline. This consistency may be established based on the following:
  If the historical and screening ALT and AST values are both ≤1.5 × ULN, there is no limit to the difference between the values.
  Patients who do not have historical ALT and AST evaluations available will have their ALT and AST repeated during the screening period to help to establish no worsening of >30% (both assessments during screening period) with >2 weeks between assessments. The second ALT, AST assessment may take place after the screening MRI-PDFF.
  If the historic ALT/AST are >1.5× elevated and screening ALT and AST are markedly improved (>50% decreased or normalized) relative to historic data, then a third ALT/AST determination will be made during screening to help establish a stable baseline.
  If at least one of the values is >1.5 × ULN and the second value is greater than the first value, the difference in the mean of ALT and AST values must be ≤30%. If the second value is greater than the first value by >30%, a third value assessed >2 weeks after the second value should be determined to help establish a lack of worsening trend in ALT/AST. If a worsening trend is confirmed (three consecutive worsening values with difference from first value and second value >30% and difference between second and third value >30%), the patient will be deemed a screen failure, but may be rescreened if ALT and AST stabilize.
15. Use of pioglitazone >15 mg d^−1^. Pioglitazone is allowed at doses up to 15 mg d^−1^ if the patient has been on stable dose for ≥12 weeks before randomization.
16. Platelet count <140,000 mm^−3^. Patients with platelets <140,000 and ≥100,000 mm^−3^ are eligible if Fib-4 < 3.5.
17. History of biliary diversion.
18. Uncontrolled hypertension (either treated or untreated) defined as systolic blood pressure ˃170 mm Hg or a diastolic blood pressure ˃100 mm Hg at screening.
19. New York Heart Association Class III or IV heart failure or known left ventricular ejection fraction <30%.
20. Uncontrolled cardiac arrhythmia.
21. At screening, confirmed QT interval corrected using Fridericia’s formula (QTcF) > 450 ms for males and >470 ms for females based on triplicate ECG assessment. At least two of the three ECGs must show a prolongation and the average of the three ECGs must be prolonged to meet criteria for exclusion. Prolonged QTcF may be repeated and confirmed following machine calibration if needed. Note: patients with bundle branch block or other conditions in which a QTcF cannot be calculated are allowed.
22. Myocardial infarction, unstable angina, percutaneous coronary intervention, coronary artery bypass graft or stroke within 12 weeks before randomization.
23. Use of illicit intravenous drugs within 5 years before randomization or a urine drug screen result positive for amphetamines, barbiturates, benzodiazepines, cocaine, methadone, opiates or phencyclidine at screening, unless a prescribed drug accounts for the positive test.
24. Active, serious medical disease with a likely life expectancy <2 years.
25. Participation in an investigational new drug trial in the 60 d or five half-lives, whichever is longer, before randomization. Patients previously treated with NASH therapeutics in an investigational trial are allowed if follow-up liver biopsy at the end of trial continued to show active NASH fibrosis meeting eligibility criteria and they have been off the NASH therapeutic for at least 24 weeks before expected randomization. If a potential NASH therapeutic studied revealed no safety issues, and in fact was not a NASH therapeutic (no effect on liver biopsy compared to placebo), participation may occur 60 d or five half-lives, whichever is longer, after discontinuation of the therapy.
26. History of major surgery (surgery involving a risk to the life of the patient; specifically, an operation upon an organ within the cranium, chest, abdomen or pelvic cavity) within 6 weeks before randomization.
27. History of cancer within the last 5 years (other than treated and believed to be cured basal or squamous cell carcinoma of the skin or resected carcinoma of the cervix).
28. Any other condition which, in the opinion of the investigator, would impede compliance, hinder completion of the study, compromise the well-being of the patient or interfere with the study outcomes.
29. Known immunocompromised status, including but not limited to individuals who have undergone organ transplantation, who are known to be positive for HIV or who have recurrent or chronic systemic bacterial, fungal, viral or protozoal infections.
30. Hypersensitivity to resmetirom or to any of the excipients or to placebo.

MAESTRO-NAFLD-1 was conducted in accordance with the ethical principles of the Declaration of Helsinki and was consistent with the International Conference on Harmonization Good Clinical Practice and applicable regulatory requirements. An institutional review board (IRB) or independent ethics committee at each site approved the protocol and all amendments (central IRB WCG, tracking no. 20192651; local IRB Duke University Health System tracking no. Pro00104842; local IRB Cedars-Sinai Office of Research Compliance and Quality Assurance tracking no. STUDY00001571). All patients provided written informed consent before enrollment.

### Randomization

As specified in the protocol, the first 30 patients were enrolled in the OL 100 mg resmetirom arm (including any patients taking thyroxine >75 μg d^−1^) before initiation of 1:1:1:1 randomization to the three DB arms (100 mg resmetirom, 80 mg resmetirom or placebo) or the OL 100 mg resmetirom arm. Randomization to the OL arm was discontinued when the target number of patients was achieved (1 July 2020). Thereafter, eligible patients were randomized 1:1:1 to the three DB arms (100 mg resmetirom, 80 mg resmetirom or placebo). Randomization was stratified by type 2 diabetes status (presence/absence) and by history of documented atherosclerotic CV disease (ASCVD; yes/no). An interactive voice and web response system was used to assign treatment. Patients and study personnel administering the study drug and performing the clinical assessments were blinded to the individual patient’s treatment (resmetirom or placebo). Select individuals were not blinded to patients’ treatment assignments to facilitate operations (for example, to prepare data monitoring committee (DMC) materials); these individuals were not otherwise involved in the study. Results of several laboratory tests, defined in the laboratory manual (for example, SHBG, lipids and FT4), were blinded to study personnel and investigators during the study to preserve the blind. Reporting of serious AEs that met the criteria for expedited reporting was also managed by the pharmacovigilance team to maintain the blind for the rest of the study team. In addition, certain laboratory results that could unblind the study and were not required for patient management were blinded.

Patients completing MAESTRO-NAFLD-1 were eligible to enroll in a 52-week OL extension (MAESTRO-NAFLD-OLE), which will provide additional data to characterize the safety and efficacy of resmetirom in adults with NAFLD/presumed NASH. In addition to the four arms described above, the MAESTRO-NAFLD-1 trial included three additional OL active treatment arms in patients with (1) noncirrhotic NASH, enrolled after the randomization period; (2) well-compensated (Child–Pugh A) NASH cirrhosis; and (3) moderate renal impairment. The OL arms in patients with at-risk NASH (enrolled after randomization period), well-compensated NASH cirrhosis and moderate renal impairment were completed after the main study and will be reported separately.

### Procedures

For all patients randomized to resmetirom treatment, dose adjustments could be triggered by an unblinded monitor. At week 12, the resmetirom dose was reduced by 20 mg if FT4 levels decreased from baseline by ≥30% (to <0.7 ng dl^−1^) on two consecutive visits (at weeks 4 and 8). In patients assigned to 100 mg resmetirom who were dose reduced to 80 mg at week 12, if FT4 levels continued to decrease from baseline by ≥30% (to <0.7 ng dl^−1^), the dose was further decreased to 60 mg at week 24. After week 24, no further resmetirom dose adjustments were permitted.

A DMC, which met at least quarterly throughout the MAESTRO-NAFLD-1 trial, evaluated the safety and key pharmacodynamic data to recommend whether the trial should continue, be modified or stopped. The DMC reviewed the unblinded safety data, including TEAEs, serious TEAEs and pharmacodynamic data. Blinded hepatic and CV adjudication committees evaluated potential liver or major adverse CV events.

A TEAE was defined as any AE with onset or post-dose worsening of any pre-existing AE (existing before the first dose of study drug), either by severity or by study drug relationship, on or after the date of the first dose of study drug up to 30 d after the date of the last dose of study drug. Patients who discontinued treatment but remained in the trial could have their safety data defined by new AEs that occurred >30 d after last dose censored (considered not treatment-emergent). AEs were coded using MedDRA v.24.0.

MRI-PDFF and MRE (at participating sites) were performed at baseline (before initiation of study drug), week 16 and week 52. MRI-PDFF was calculated using a proton density weighted two-dimensional multi-echo gradient echo pulse sequence, including six nominally in- and opposed-phase echoes. The reconstruction accounted for T2*-related signal decay and the spectral complexity of fat. MRE was obtained and reconstructed using vendor-supplied hardware and software. FibroScans were obtained at prescreening or during screening and at week 52. Both MRI and MRE image analyses were conducted using OsiriX 9 (Pixmeo Sarl). Vital signs, a 12-lead ECG and clinical laboratory testing (hematology, chemistry and urinalysis) and assessment of lipid parameters, thyroid hormone parameters and other biomarkers were conducted at specified visits.

Liver volume was assessed at baseline and week 52 in the OL 100 mg resmetirom arm. Volumes were estimated based on motion-robust single-shot fast-spin echo MRI obtained as part of the MRI examinations for MRI-PDFF estimation. Images were obtained in the axial plane with a slice thickness of 6–8 mm and inter-slice gap of 0–2 mm, with maximum in-plane voxel dimensions of 1.9 × 1.9 mm. Initial two-dimensional contours were developed using a model based on the UNet architecture. Contours were visually assessed and adjusted by imaging core laboratory technologists with experience in liver volumetry and validated by a fellowship-trained abdominal radiologist with over 15 years’ experience in abdominal radiology. Liver volumes were estimated in ml based on the number of voxels segmented, in-plane voxel size and section spacing.

### Objectives

The primary objective was to evaluate the safety and tolerability of resmetirom as measured by the incidence of TEAEs over 52 weeks of treatment. The same objective applied to the OL noncirrhotic NASH cohort that enrolled in the trial from the time of initiation to the closure of the 1:1:1:1 randomization (1 July 2020). Key secondary objectives were %CFB to week 24 in LDL-C; %CFB to week 24 in apoB; %CFB to week 16 in hepatic fat (measured by MRI-PDFF); %CFB to week 24 in TGs in the subgroup with baseline TGs ≥ 150 mg dl^−1^; CFB to week 52 in FibroScan CAP; and CFB to week 52 in FibroScan VCTE/LSM in the subgroup with baseline VCTE/LSM ≥ 7.2 kPa.

Additional secondary objectives were %CFB to week 52 in hepatic fat (measured by MRI-PDFF); %CFB to week 24 in LDL-C in the subgroup with baseline LDL-C ≥ 100 mg dl^−1^, apoB in the subgroup with baseline LDL-C ≥ 100 mg dl^−1^, Lp(a) in the subgroup with baseline Lp(a) >10 nmol l^−1^, apoCIII, HDL-C, RLP cholesterol, VLDL cholesterol and VLDL and chylomicron TG; %CFB to week 48 in LDL-C, LDL-C in the subgroup with baseline LDL-C ≥ 100 mg dl^−1^, apoB, apoB in the subgroup with baseline LDL-C ≥ 100 mg dl^−1^, TGs in the subgroup with TGs ≥ 150 mg dl^−1^, Lp(a) in the subgroup with Lp(a) >10 nmol l^−1^, apoCIII, HDL-C, RLP cholesterol, VLDL cholesterol and VLDL and chylomicron TGs; CFB to week 52 in liver enzymes (ALT, AST and GGT) in the subgroup with baseline ALT ≥ 30 IU l^−1^; CFB to week 52 in CK-18, adiponectin, ELF in the subgroup with baseline ELF ≥ 9.8, amino-terminal propeptide of type III procollagen (PIIINP) in the subgroup with baseline PIIINP ≥ 9 ng ml^−1^, TIMP-1 in the subgroup with baseline TIMP-1 ≥ 240 ng ml^−1^ and hyaluronic acid in the subgroup ≥50 ng ml^−1^. PROs were assessed by the chronic liver disease questionnaire at week 52.

Exploratory outcomes included change from baseline to week 52 in sex hormones (estradiol, follicle-stimulating hormone, luteinizing hormone, testosterone, free testosterone and SHBG); CFB to week 52 in thyroid hormones (FT3, FT4, rT3, FT3/rT3, TSH and thyroxine-binding globulin).

### Statistical analysis

**Extended Data Fig. 4:**
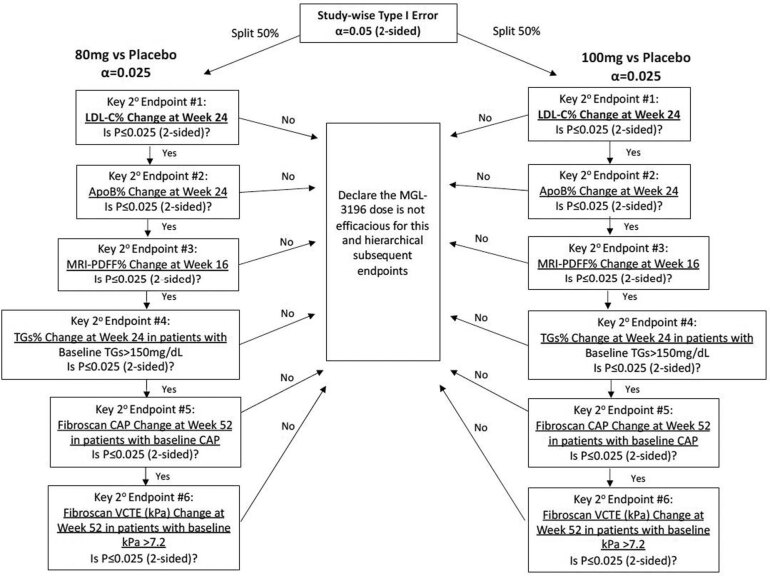
Hierarchical testing procedure. Hierarchical testing procedure for key secondary end points.

Sample size was based on safety and regulatory considerations to facilitate the evaluation of the treatment effect within subgroups of interest. For evaluation of the key secondary end points, randomizing ≥200 patients to each of the three DB arms was expected to provide >90% power to demonstrate a statistically significant difference between each resmetirom dose and placebo at the two-sided 0.025 significance level in the %CFB in LDL-C at week 24, assuming a ≥13.5% difference between the resmetirom arm and placebo arm with a within-treatment s.d. of 16%. Other key secondary lipid end points and percent change in hepatic fat between the resmetirom and placebo arms had ≥90% power. This trial was designed to maintain an overall study-wise type I error rate of α = 0.05 for the key secondary end points only. The error rate was controlled by first splitting the overall two-sided α = 0.05 into two partitions via the Bonferroni method and then the key secondary end points were tested in a prespecified hierarchical order (Extended Data Fig. 4). Customary usage of s.d. or s.e.m. was applied to statistical outputs. For population characteristics such as baseline measurements, the s.d. was used to describe the variability. As a measure of dispersion around the mean, the s.d. allows us to best understand the characteristics of the population being studied. When describing changes from baseline or %CFB, for example in lipid measurements, the s.e.m. was used. The s.e.m. enables an understanding of the magnitude of change observed in the sample studied. If any of the statistical comparisons were not statistically significant, then all subsequent tests were considered regular secondary end points that were not controlled for multiple testing. Subsequent secondary and exploratory end points were not controlled for multiplicity and *P* values were considered nominal.

The primary analyses were conducted after all patients in the DB arms completed the 52-week treatment period and 4-week follow-up period (or discontinued from the trial with a 4-week follow-up period). TEAEs were analyzed descriptively for each treatment arm. For the primary analysis of key secondary lipid end points, an ANCOVA model was used to analyze each set of ten imputed datasets. The ANCOVA models for each lipid outcome included %CFB to week 24 (and all other end point visits) as the dependent variable; treatment and stratification factors (presence of type 2 diabetes and ASCVD) as independent variables; and baseline lipid values as a covariable. The statistical method of estimating the treatment effect and testing for apoB and TGs was similar to the LDL-C end point; a similar ANCOVA model was used whereby the baseline value of the dependent variable was included as covariate instead of LDL-C.

This study was impacted by COVID-19 and the estimand in the statistical analysis plan reflected the statistical approach taken to address COVID-19-related missing data. The strategy was used to evaluate the effect of resmetirom as though the blister pack kit shortage due to the COVID-19 pandemic or other missing receipt of study drug (due to site closures or other COVID-19-related missed visits) had not happened. To address the COVID-19 impact, the week-24 lipid assessment and lipid assessment at any visit in which fasting lipids were measured (weeks 4, 12, 16, 20, 24, 28, 32, 36, 40, 44, 48 and 52) that was impacted by the blister pack kit shortage at the previous visit (unavailability of study drug during the 4 weeks preceding the visit), was considered missing. Per protocol and of note, all post-baseline lipids/lipoproteins and MRI-PDFF measurements were blinded during the study, as was the treatment code.

The analysis of lipids was performed after imputation was performed for missing results. The imputation was conducted in two stages, where stage 1 utilized a single imputation using a patient’s own data and stage 2 used a multiple imputation approach. In stage 1, invalid and missing data caused by the COVID-19 pandemic were imputed using the ‘valid visit’ lipid measurement (if available) obtained just before the missing visit. If the previous visit was missing or invalid, the lipid value obtained immediately after the missing visit was imputed for the missing visit. For lipid data that were still missing after the single imputation approach described above, those missing data were imputed using the non-missing lipid values (including the singly imputed data from above) based on missing-at-random-based multiple imputation. When applying the missing-at-random-based multiple imputation, data were imputed separately by randomized treatment group and randomization stratification factors. Lipid assessments impacted by other COVID-19-related factors including drop-out from the study due to COVID-19 (including drop outs caused by blister pack kit shortages) and nonadherence to dosing due to COVID-19 were not considered for single imputation given that they were either infrequent (missed visits) or difficult to track. The values for lipid end points affected by all other intercurrent events (COVID-19-related and non-COVID-19-related) were multiply imputed as described in the statistical analysis plan.

In this trial, few patients had a baseline FibroScan VCTE LSM that met criteria for analysis. As such, a responder analysis was performed to evaluate the percent of patients whose FibroScan VCTE LSM improved or worsened by either ≥2 kPa or ≥30% from baseline at week 52. A similar responder analysis was performed for MRE as well to evaluate the percent of patients whose LSM by MRE improved or worsened by ≥19% from baseline at week 52. For analysis of the OL resmetirom arm, end points were summarized descriptively and analyzed by a within-group CFB. For descriptive statistical summaries, the number of non-missing observations, mean, s.d., median, minimum and maximum were calculated for continuous variables. For categorical variables, the frequency and percentage of each category were provided. All statistical analyses were performed with SAS v.9.4 or above.

Safety end points were evaluated in the safety population, which included all randomized patients who received ≥1 dose of the DB study drug. Efficacy end points were evaluated in the mITT population, which included all randomized patients who received ≥1 dose of the DB study drug and had a baseline and ≥1 post-baseline measurement. The ITT population, which included all DB randomized patients, could be used for sensitivity analysis if it differed substantially from the mITT population.

### Protocol deviations resulting from COVID-19

The study was conducted between December 2019 and December 2021 at 79 sites in the United States during the height of the COVID-19 pandemic. On 27 March 2020, Madrigal, the sponsor, issued an administrative letter (following the Conduct of Clinical Trials of Medical Products During the COVID-19 Public Health Emergency Guidance for Industry, Investigators and IRBs issued by the US Food and Drug Administration on 25 March 2020) to sites with information on the following topics:

- Documentation of protocol deviations with attribution to COVID-19 when applicable;
- Flexibility for screening and enrollment windows, study visit windows and study procedures and collection of laboratory results at study visits in situations in which patient access was unsafe or restricted due to COVID-19, including allowing telehealth phone visits;
- Delivery of investigational medicinal product to patients who could not come to the investigative site for a study visit;
- Guidance on investigative site monitoring, focusing on a remote site monitoring plan; and
- Changes in informed consent to implement a more flexible approach to clinical studies.

Largely due to COVID-19-related drug kit delays in the DB arms, 86–88% of DB patients missed study visits (inclusive of visits where no study drug was provided) and the average number of missed monthly visits in the DB arms was 2–3 over the 52-week treatment period. Only 19% of OL non-cirrhotic (NC) patients with NASH had missed study visits.

### Reporting summary

Further information on research design is available in the Nature Portfolio Reporting Summary linked to this article.

## Online content

Any methods, additional references, Nature Portfolio reporting summaries, source data, extended data, supplementary information, acknowledgements, peer review information; details of author contributions and competing interests; and statements of data and code availability are available at 10.1038/s41591-023-02603-1.

### Supplementary information

Supplementary Information Supplementary Tables 1–4

Reporting Summary

## Figures and Tables

**Extended Data Table 1:**
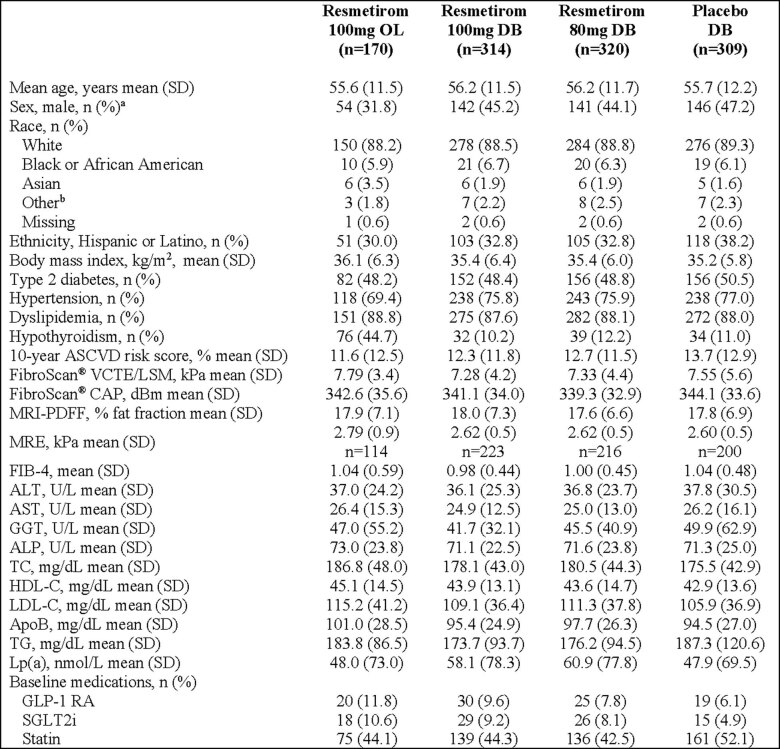
Demographic and baseline characteristics (modified intent-to-treat population)

^a^Sex was self-reported by the patient.

^b^Includes American Indian or Alaska Native; Native Hawaiian or other Pacific Islander; and Other. ALP, alkaline phosphatase; ALT, alanine aminotransferase; apoB, apolipoprotein B; ASCVD, atherosclerotic cardiovascular disease; AST, aspartate aminotransferase; CAP, controlled attenuation parameter; DB, double-blind; FIB-4, fibrosis-4; GGT, gamma-glutamyl transferase; GLP-1 RA, glucagon-like peptide-1 receptor agonist; HDL-C, high-density lipoprotein cholesterol; LDL-C, low-density lipoprotein cholesterol; LSM, liver stiffness measurement; MRE, magnetic resonance elastography; MRI-PDFF, magnetic resonance imaging-proton density fat fraction; OL, open-label; SD, standard deviation; TC, total cholesterol; TG, triglycerides; VCTE, vibration-controlled transient elastography.

**Extended Data Table 2:**
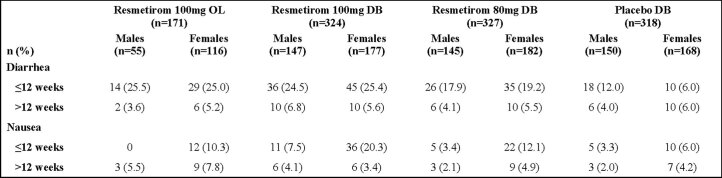
Gastrointestinal adverse events by sex and time (safety population)

DB, double-blind; OL, open-label.

**Extended Data Table 3:**
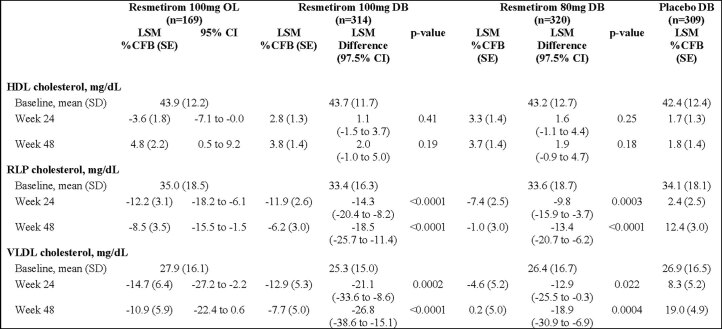
Lipids and lipoproteins at baseline and percent change from baseline^a^

^a^End points reported as percent change from baseline (%CFB). Lipids, lipoprotein particles and lipoproteins are evaluated at Week 24 and Week 48 as LSM %CFB; other analytes as Week 52 LSM CFB. This trial was designed to maintain an overall study-wise type I error rate of α = 0.05 for the key secondary end points only. The error rate was controlled by first splitting the overall two-sided α = 0.05 into 2 partitions via the Bonferroni method, and then the key secondary end points tested in a prespecified hierarchical order. For the primary analysis of key secondary lipid end points, an analysis of covariance (ANCOVA) model was used to analyze each set of 10 imputed datasets. The ANCOVA models for each lipid outcome included percent change from baseline to Week 24 (and all other end point visits) as the dependent variable; treatment and stratification factors (presence of type 2 diabetes and ASCVD) as independent variables; and baseline lipid values as a covariable. The statistical method of estimating the treatment effect and testing for apoB and TG was similar to the LDL-C end point; a similar ANCOVA model was used whereby the baseline value of the dependent variable was included as covariate instead of LDL-C.CFB, change from baseline; CI, confidence interval; DB, double-blind; ELF, enhanced liver fibrosis; HDL, high-density lipoprotein; HDL-C, high-density lipoprotein cholesterol; LDL, low-density lipoprotein; LSM, least squares mean; OL, open-label; RLP, remnant-like particle; SD, standard deviation; SE, standard error VLDL, very low-density lipoprotein.

**Extended Data Table 4:**
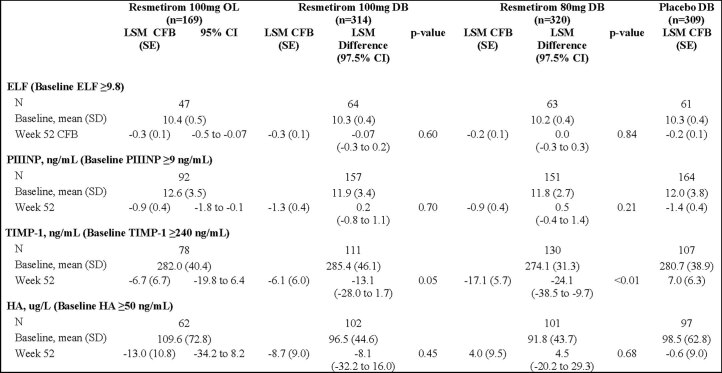
ELF test and ELF components change from baseline

This trial was designed to maintain an overall study-wise type I error rate of α = 0.05 for the key secondary end points only. The error rate was controlled by first splitting the overall two-sided α = 0.05 into 2 partitions via the Bonferroni method, and then the key secondary end points tested in a prespecified hierarchical order. For the primary analysis of key secondary lipid end points, an analysis of covariance (ANCOVA) model was used to analyze each set of 10 imputed datasets. A similar ANCOVA model was used whereby the baseline value of the dependent variable was included as covariate instead of LDL-C.CFB, change from baseline; CI, confidence interval; DB, double-blind; ELF, enhanced liver function; PIIINP, amino-terminal propeptide of type III collagen; TIMP, tissue mettaloproteinase-1; HA, hyaluronic acid

**Extended Data Table 5:**
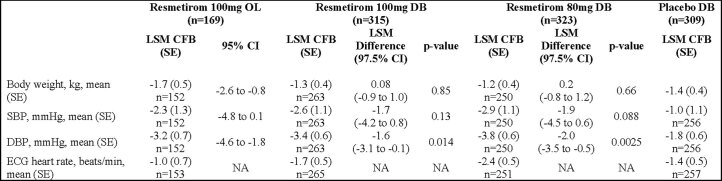
Change from baseline in metabolic factors at Week 52 (safety population)

CFB, change from baseline; CI, confidence interval; DB, double-blind; DBP, diastolic blood pressure; ECG, electrocardiogram; LSM, least squares mean; NA, not available; OL, open-label; SEM, standard error of the mean; SBP, systolic blood pressure; SE, standard error.

**Extended Data Table 6:**
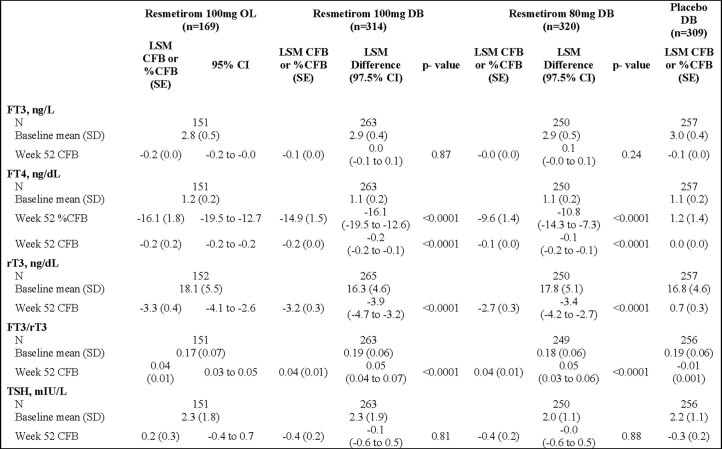
Thyroid hormones at baseline and change from baseline^a^ at Week 52 (observed data)

^a^Free thyroxine (FT4) reported as both change from baseline (CFB) and percent change from baseline (%CFB) at Week 52. All other thyroid hormones reported as change from baseline (CFB) at Week 52. This trial was designed to maintain an overall study-wise type I error rate of α = 0.05 for the key secondary endpoints only. The error rate was controlled by first splitting the overall two-sided α = 0.05 into 2 partitions via the Bonferroni method, and then the key secondary endpoints tested in a prespecified hierarchical order. An ANCOVA model was used whereby the baseline value of the dependent variable was included as covariate instead of LDL-C CFB, change from baseline; CI, confidence interval; DB, double-blind; LSM, least squares mean; OL, open-label; RT3, reverse triiodothyronine; SE, standard error; FT3, free triiodothyronine; FT4, free thyroxine; TSH, thyroid-stimulating hormone.

## Data Availability

Datasets generated as part of the MAESTRO-NAFLD-1 trial are considered commercially sensitive and as such, are not publicly available. Requests for data supporting findings in this manuscript should be made to the corresponding author (S.A.H.). Data may be shared in the form of aggregate data summaries and via a data transfer agreement 3 years after publication. Individual patient-level data are subject to patient privacy and cannot be shared.
