# Supplementary material for: Resmetirom for nonalcoholic fatty liver disease: a randomized, double-blind, placebo-controlled phase 3 trial
Source: Nat Med. 2023 Oct 16;29(11):2919–28. doi: 10.1038/s41591-023-02603-1 (PMC10667098; doi:10.1038/s41591-023-02603-1)
Supplement: Supplementary file 1 — Supplementary Tables 1–4 [file 41591_2023_2603_MOESM1_ESM.pdf]

nature medicine

Article

https://doi.org/10.1038/s41591-023-02603-1

Resmetirom for nonalcoholic fatty liver disease: a randomized, double-blind, placebo-controlled phase 3 trial

In the format provided by the authors and unedited

Supplemental Data Tables

Supplement Data Table 1. Sex hormones at baseline and change from baseline[a] at Week 52 (observed data)

	Resmetirom 100mg OL (n=169)		Resmetirom 100mg DB (n=314)			Resmetirom 80mg DB (n=320)			Placebo DB (n=309)
	LSM CFB or %CFB (SE)	95% CI	LSM CFB or %CFB (SE)	LSM Difference (97.5% CI)	p-value	LSM CFB or %CFB (SE)	LSM Difference (97.5% CI)	p-value	LSM CFB or %CFB (SE)
**Estradiol, ng/L (female)**									
N	105		145			138			127
Baseline mean (SD)	35.7 (58.7)		41.9 (66.5)			37.4 (70.0)			38.6 (59.6)
Week 52 CFB (SE)	11.0 (8.8)	-6.3 to 28.4	7.5 (8.2)	9.5 (-9.5 to 28.6)	0.26	5.0 (8.3)	7.1 (-12.2 to 26.3)	0.41	-2.1 (8.4)
**Estradiol, ng/L (male)**									
N	46		118			111			129
Baseline mean (SD)	28.4 (10.7)		34.3 (14.2)			35.9 (18.5)			32.8 (14.9)
Week 52 CFB (SE)	11.7 (2.2)	7.4 to 15.9	8.9 (1.5)	10.4 (6.8 to 14.1)	<0.0001	3.3 (1.5)	4.8 (1.1 to 8.6)	0.0037	-1.5 (1.5)
**FSH, IU/L (female)**									
N	105		145			138			126
Baseline mean (SD)	36.4 (25.7)		40.0 (27.5)			41.5 (25.7)			38.0 (25.2)
Week 52 CFB (SE)	1.3 (1.2)	-1.0 to 3.6	1.6 (1.1)	-0.4 (-2.9 to 2.1)	0.72	0.9 (1.1)	-1.1 (-3.6 to 1.5)	0.35	2.0 (1.1)
**FSH, IU/L (male)**									
N	46		118			111			129
Baseline mean (SD)	6.3 (3.3)		6.3 (5.2)			6.6 (7.1)			6.5 (5.9)

Week 52 CFB (SE)	0.6 (0.4)	-0.2 to 1.3	0.6 (0.3)	0.5 (-0.2 to 1.1)	0.12	0.3 (0.3)	0.2 (-0.5 to 0.8)	0.58	0.1 (0.3)
**LH, IU/L (female)**									
n	105		143			138			126
Baseline mean (SD)	21.1 (12.8)		23.6 (13.6)			24.6 (14.1)			22.1 (12.9)
Week 52 CFB (SE)	-0.0 (0.9)	-1.9 to 1.8	1.2 (0.9)	-0.5 (-2.5 to 1.6)	0.61	0.8 (0.9)	-0.9 (-2.9 to 1.2)	0.34	1.7 (0.9)
**LH, IU/L (male)**									
n	46		118			111			129
Baseline mean (SD)	5.6 (3.0)		5.8 (3.0)			5.8 (5.1)			6.0 (3.5)
Week 52 CFB (SE)	0.3 (0.4)	-0.5 to 1.2	0.9 (0.3)	0.8 (0.1 to 1.6)	0.01	0.4 (0.3)	0.4 (-0.4 to 1.1)	0.28	0.1 (0.3)
**Testosterone, ug/L (female)**									
n	105		145			138			127
Baseline mean (SD)	0.13 (0.14)		0.17 (0.25)			0.18 (0.30)			0.17 (0.19)
Week 52 CFB (SE)	0.1 (0.0)	0.0 to 0.1	0.1 (0.0)	0.1 (0.1 to 0.2)	<0.0001	0.1 (0.0)	0.1 (0.1 to 0.2)	<0.0001	0.0 (0.0)
**Testosterone, ug/L (male)**									
n	46		118			111			129
Baseline mean (SD)	3.7 (1.6)		3.6 (1.5)			3.8 (2.5)			3.6 (1.7)
Week 52 CFB (SE)	2.8 (0.4)	2.1 to 3.5	2.0 (0.3)	2.1 (1.4 to 2.7)	<0.0001	1.4 (0.3)	1.5 (0.9 to 2.2)	<0.0001	-0.1 (0.3)
**Free testosterone, ug/L (female)**									
n	75		112			96			91
Baseline mean (SD)	0.0031 (0.0029)		0.0039 (0.0059)			0.0038 (0.0048)			0.0035 (0.0028)
Week 52 CFB (SE)	-0.0006 (0.0004)	-0.0015 to 0.0003	-0.0003 (0.0004)	-0.0006 (-0.0014 to 0.0003)	0.12	0.0004 (0.0004)	0.0002 (-0.0007 to 0.0011)	0.61	0.0002 (0.0004)
**Free testosterone, ug/L (male)**									

n	45		115			108			127
Baseline mean (SD)	0.0690 (0.0218)		0.0718 (0.0315)			0.0758 (0.0498)			0.0713 (0.0370)
Week 52 CFB (SE)	0.024 (0.0054)	-0.0081 to 0.0130	-0.0009 (0.0038)	0.0029 (-0.0061 to 0.0120)	0.48	0.0077 (0.0037)	0.0115 (0.0023 to 0.0208)	0.0005 4	-0.0038 (0.0036)

[a]Sex hormones reported as change from baseline (CFB) at Week 52.

CFB, change from baseline; CI, confidence interval; DB, double-blind; FSH, follicle-stimulating hormone; LH, luteinizing hormone; LSM, least squares mean; OL, open-label; SD, standard deviation; SE, standard error; SHBG, sex hormone binding globulin.

Supplement Data Table 2. Sex hormone binding globulin (SHBG) at baseline and change from baseline[a] at Week 52 (observed data)

	**Resmetirom 100mg OL (n=169)**		**Resmetirom 100mg DB (n=314)**			**Resmetirom 80mg DB (n=320)**			**Placebo DB (n=309)**
	**LSM CFB or %CFB (SE)**	**95% CI**	**LSM CFB or %CFB (SE)**	**LSM Difference (97.5% CI)**	**p-value**	**LSM CFB or %CFB (SE)**	**LSM Difference (97.5% CI)**	**p-value**	**LSM CFB or %CFB (SE)**
**SHBG, nmol/L**									
N	151		263			248			255
Baseline mean (SD)	37.7 (24.4)		35.8 (19.8)			35.8 (20.7)			35.1 (18.7)
Week 52 %CFB (SE)	182.6 (13.0)	157.0 to 208.1	161.1 (11.0)	157.5 (131.5 to 183.4)	<0.0001	99.6 (10.9)	96.0 (69.6 to 122.4)	<0.0001	3.6 (10.9)
Week 52 CFB (SE)	60.3 (4.4)	51.7 to 68.9	53.0 (3.7)	52.4 (43.6 to 61.2)	<0.0001	32.7 (3.7)	32.1 (23.2 to 41.0)	<0.0001	0.6 (3.7)
**SHBG, nmol/L (female)**									
N	105		145			137			126
Baseline mean (SD)	38.7 (26.0)		38.9 (22.7)			40.7 (24.1)			36.8 (20.5)
Week 52 %CFB (SE)	199.3 (19.6)	160.8 to 237.8	197.6 (18.2)	184.0 (141.5 to 226.5)	<0.0001	133.0 (18.4)	119.5 (76.3 to 162.6)	<0.0001	13.5 (18.7)
Week 52 CFB (SE)	68.6 (6.6)	55.5 to 81.4	65.3 (6.1)	61.8 (47.5 to 76.1)	<0.0001	43.3 (6.2)	39.9 (25.4 to 54.4)	<0.0001	3.5 (6.3)
**SHBG, nmol/L (male)**									
N	46		118			111			129
Baseline mean (SD)	35.5 (20.2)		32.1 (14.8)			29.8 (13.4)			33.5 (16.6)
Week 52 %CFB (SE)	156.0 (14.8)	126.9 to 185.1	122.9 (10.5)	125.5 (100.4 to 150.5)	<0.0001	67.1 (10.2)	69.7 (44.1 to 95.2)	<0.0001	-2.6 (10.0)
Week 52 CFB (SE)	44.7 (5.0)	34.8 to 54.6	39.2 (3.6)	41.7 (3.8) (33.2 to 50.2)	<0.0001	21.9 (3.5)	24.5 (3.9) (15.8 to 33.2)	<0.0001	-2.5 (3.4)

[a] Sex hormone binding globulin (SHBG) reported as both change from baseline (CFB) and percent change from baseline (%CFB) at Week 52.
CFB, change from baseline; CI, confidence interval; DB, double-blind; LSM, least squares mean; OL, open-label; SE, standard error; SHBG, sex hormone binding globulin.

Supplement Data Table 3. Thyroid hormones at baseline and change from baseline[a] at Week 52 (observed data)

	Resmetirom 100mg OL (n=169)		Resmetirom 100mg DB (n=314)			Resmetirom 80mg DB (n=320)			Placebo DB (n=309)
	**LSM CFB or %CFB (SE)**	**95% CI**	**LSM CFB or %CFB (SE)**	**LSM Difference (97.5% CI)**	**p-value**	**LSM CFB or %CFB (SE)**	**LSM Difference (97.5% CI)**	**p-value**	**LSM CFB or %CFB (SE)**
**FT3, ng/L (not on thyroxine)**									
n	81		234			217			231
Baseline mean (SD)	3.0 (0.4)		3.0 (0.4)			2.9 (0.4)			3.0 (0.4)
Week 52 CFB	-0.1 (0.1)	-0.2 to 0.0	-0.1 (0.0)	0.0 (-0.1 to 0.1)	0.84	-0.1 (0.0)	0.1 (-0.0 to 0.2)	0.19	-0.1 (0.0)
**FT3, ng/L (thyroxine-treated)**									
n	70		29			33			26
Baseline mean (SD)	2.7 (0.6)		2.6 (0.4)			2.9 (0.7)			2.7 (0.4)
Week 52 CFB	-0.1 (0.1)	-0.3 to 0.0	-0.0 (0.1)	-0.0 (-0.3 to 0.3)	0.98	0.0 (0.1)	0.0 (-0.2 to 0.3)	0.80	-0.0 (0.1)
**FT4, ng/dL (not on thyroxine)**									
n	81		234			217			231
Baseline mean (SD)	1.1 (0.2)		1.1 (0.2)			1.1 (0.2)			1.1 (0.2)
Week 52 %CFB	-18.9 (2.2)	-23.3 to -14.5	-14.3 (1.6)	-15.4 (-19.0 to -11.7)	<0.0001	-9.9 (1.5)	-11.0 (-14.7 to -7.3)	<0.0001	1.1 (1.6)
Week 52 CFB	-0.2 (0.0)	-0.3 to -0.2	-0.2 (0.0)	-0.2 (-0.2 to -0.1)	<0.0001	-0.1 (0.0)	-0.1 (-0.2 to -0.1)	<0.0001	0.0 (0.0)
**FT4, ng/dL (thyroxine-treated)**									
n	70		29			33			26
Baseline mean (SD)	1.3 (0.2)		1.1 (0.2)			1.3 (0.3)			1.2 (0.2)
Week 52 %CFB	-14.8 (3.0)	-20.8 to -8.8	-19.2 (3.8)	-23.0 (-33.5 to -12.5)	<0.01	-6.4 (3.9)	-10.2 (-20.3 to 0.0)	0.0251	3.8 (3.9)
Week 52 CFB	-0.2 (0.0)	-0.3 to -0.1	-0.2 (0.0)	-0.3 (-0.4 to -0.1)	<0.0001	-0.1 (0.0)	-0.1 (-0.3 to -0.0)	0.0118	0.0 (0.0)

**rT3, ng/dL (not on thyroxine)**									
n	82			236			217		231
Baseline mean (SD)	16.5 (5.1)			16.1 (4.2)			17.5 (4.9)		16.5 (4.4)
Week 52 CFB	-3.5 (0.5)	-4.4 to -2.6	-2.9 (0.3)	-3.8 (-4.5 to -3.0)	<0.0001	-2.5 (0.3)	-3.3 (-4.1 to -2.6)	<0.0001	0.9 (0.3)
**rT3, ng/dL (thyroxine-treated)**									
n	70			29			33		26
Baseline mean (SD)	20.1 (5.3)			18.1 (6.8)			19.6 (5.6)		18.6 (5.5)
Week 52 CFB	-3.9 (0.8)	-5.5 to -2.2	-4.9 (1.0)	-5.5 (-8.3 to -2.7)	<0.0001	-3.8 (1.0)	-4.4 (-7.1 to -1.7)	0.0004	0.6 (1.0)
**FT3/rT3 (not on thyroxine)**									
n	81			234			216		230
Baseline mean (SD)	0.20 (0.006)			0.20 (0.006)			0.18 (0.006)		0.19 (0.006)
Week 52 CFB	0.05 (0.01)	0.03 to 0.07	0.04 (0.01)	0.05 (0.04 to 0.07)	<0.0001	0.04 (0.01)	0.05 (0.04 to 0.006)	<0.0001	-0.01 (0.01)
**FT3/rT3 (thyroxine-treated)**									
n	70			29			33		26
Baseline mean (SD)	0.14 (0.07)			0.16 (0.05)			0.16 (0.07)		0.16 (0.06)
Week 52 CFB	0.03 (0.01)	0.01 to 0.05	0.04 (0.01)	0.04 (0.01 to 0.07)	<0.0023	0.03 (0.01)	0.03 (0.00 to 0.06)	0.0136	0.00 (0.01)
**TSH, mIU/L (not on thyroxine)**									
n	81			234			217		230
Baseline mean (SD)	2.2 (1.3)			2.2 (1.3)			2.0 (1.1)		2.2 (1.0)
Week 52 CFB	-0.2 (0.1)	-0.4 to -0.0	-0.3 (0.1)	-0.1 (-0.2 to 0.1)	0.33	-0.3 (0.1)	0.0 (-0.1 to 0.2)	0.96	-0.3 (0.1)
**TSH, mIU/L (thyroxine-treated)**									
n	70			29			33		26
Baseline mean (SD)	2.4 (2.3)			3.0 (4.4)			1.6 (1.2)		2.2 (1.6)
Week 52 CFB	0.4 (1.2)	-1.9 to 2.7	-0.6 (1.5)	0.0 (-4.0 to 4.1)	0.99	-0.9 (1.5)	-0.3 (-4.2 to 3.7)	0.89	-0.6 (1.5)
**TBG, mg/L (not on thyroxine)**									
n	82			234			214		230

Baseline mean (SD)	22.8 (4.7)		21.3 (6.4)			21.5 (6.0)			21.3 (5.3)
Week 52 CFB	0.7 (0.6)	-0.6 to 1.9	1.4 (0.5)	-0.3 (-1.3 to 0.8)	0.56	-0.5 (0.4)	-1.2 (-2.2 to -0.1)	0.01	1.7 (0.4)
**TBG, mg/L (thyroxine-treated)**									
n	70		29			33			26
Baseline mean (SD)	25.3 (7.4)		24.5 (6.0)			22.0 (7.6)			20.3 (7.2)
Week 52 CFB	-1.0 (0.8)	-2.6 to 0.6	-0.2 (1.0)	-2.4 (-5.2 to 0.5)	0.06	1.5 (1.0)	-0.6 (-3.3 to 2.1)	0.61	2.1 (1.1)

[a] Free thyroxine (FT4) reported as both change from baseline (CFB) and percent change from baseline (%CFB) at Week 52. All other thyroid hormones reported as change from baseline (CFB) at Week 52.

CFB, change from baseline; CI, confidence interval; DB, double-blind; LSM, least squares mean; OL, open-label; rT3, reverse triiodothyronine; SD, standard deviation; SE, standard error; FT3, free triiodothyronine; FT4, free thyroxine; TBG, thyroxine binding globulin; TSH, thyroid-stimulating hormone.

Supplement Data Table 4. Biomarkers, lipids, and lipoproteins at baseline and percent change from baseline[a]

	Resmetirom 100mg OL (n=169)		Resmetirom 100mg DB (n=314)			Resmetirom 80mg DB (n=320)			Placebo DB (n=309)
	**LSM %CFB or CFB (SE))**	**95% CI**	**LSM %CFB or CFB (SE))**	**LSM Difference (97.5% CI)**	**p-value**	**LSM %CFB or CFB (SE))**	**LSM Difference (97.5% CI)**	**p-value**	**LSM %CFB or CFB (SE))**
**HDL particles, umol/L**									
Baseline, mean (SD)	32.2 (6.8)		31.0 (6.3)			31.1 (6.6)			31.3 (6.6)
Week 24	0.3 (1.3)	-2.3 to 2.9	3.1 (1.1)	2.3 (-0.3 to 4.8)	0.05	2.1 (1.1)	1.3 (-1.3 to 3.9)	0.25	0.7 (1.1)
Week 48	4.3 (1.5)	1.4 to 7.2	1.6 (1.3)	3.0 (0.01 to 6.0)	0.025	-0.1 (1.3)	1.3 (-1.8 to 4.3)	0.35	-1.4 (1.3)
**HDL particle size, nm**									
Baseline, mean (SD)	8.9 (0.4)		8.9 (0.4)			8.9 (0.4)			8.8 (0.3)
Week 24	-0.0 (0.3)	-0.7 to 0.6	0.2 (0.3)	-0.0 (-0.7 to 0.6)	0.93	0.4 (0.3)	0.2 (-0.5 to 0.8)	0.55	0.2 (0.3)
Week 48	0.2 (0.4)	-0.6 to 0.9	0.1 (0.3)	0.0 (-0.7 to 0.8)	0.90	0.0 (0.3)	-0.0 (-0.8 to 0.7)	0.80	0.1 (0.3)
**IDL particles, nmol/L**									
Baseline, mean (SD)	113.9 (98.0)		95.9 (87.5)			107.8 (96.7)			103.3 (94.5)
Week 24	532.6 (244.9)	52.1 to 1013.2	96.8 (203.6)	19.5 (-458.2 to 497.1)	0.93	83.3 (201.8)	6.0 (-473.5 to 485.5)	0.98	77.3 (201.2)
Week 48	304.1 (123.1)	62.4 to 545.7	139.3 (106.8)	78.7 (-169.6 to 327.0)	0.48	62.2 (107.7)	1.6 (-250.9 to 254.1)	0.99	60.6(104.5)
**LDL particles, nmol/L**									
Baseline, mean (SD)	1347.8 (449.7)		1311.8 (394.8)			1344.7 (399.1)			1290.0 (430.8)
Week 24	-21.7 (2.1)	-25.7 to -17.6	-16.7 (1.7)	-13.1 (-17.1 to -9.0)	<0.0001	-15.5 (1.7)	-11.8 (-15.9 to -7.8)	<0.0001	-3.6 (1.7)

Week 48	-21.0 (2.3)	-25.5 to -16.5	-15.6 (2.0)	-13.5 (-18.1 to -8.9)	<0.0001	-15.1 (2.0)	-13.1 (-17.7 to -8.4)	<0.0001	-2.1 (1.9)
**LDL particle size, nm**									
Baseline, mean (SD)	20.3 (0.6)		20.3 (0.5)			20.4 (0.6)			20.2 (0.5)
Week 24	-0.3 (0.2)	-0.7 to 0.20	0.1 (0.2)	0.1 (-0.4 to 0.5)	0.78	0.1 (0.2)	0.0 (-0.4 to 0.4)	0.93	0.1 (0.2)
Week 48	0.7 (0.2)	0.2 to 1.1	0.6 (0.2)	0.3 (-0.2 to 0.7)	0.21	0.3 (0.2)	-0.0 (-0.5 to 0.4)	0.86	0.4 (0.2)
**Large HDL particles, umol/L**									
Baseline, mean (SD)	5.4 (2.5)		5.3 (2.6)			5.3 (2.7)			5.0 (2.4)
Week 24	0.8 (6.6)	-12.0 to 13.6	2.0 (5.4)	-7.5 (-20.3 to 5.2)	0.18	3.3 (5.4)	-6.3 (-19.0 to 6.5)	0.27	9.6 (5.4)
Week 48	6.2 (4.7)	-2.9 to 15.4	3.6 (4.1)	-1.30 (-10.7 to 8.1)	0.76	2.3 (4.1)	-2.6 (-12.1 to 7.0)	0.55	4.8 (4.0)
**Large LDL particles, nmol/L**									
Baseline, mean (SD)	272.7 (252.1)		304.4 (244.2)			316.6 (266.3)			263.7 (230.7)
Week 24	63.2 (87.9)	-108.8 to 236.1	96.3 (71.8)	57.0 (-105.4 to 219.3)	0.43	71.1 (72.1)	31.8 (-133.2 to 196.8)	0.67	39.3 (72.7)
Week 48	57.4 (104.5)	-147.8 to 262.5	126.6 (89.0)	29.6 (-169.2 to 228.5)	0.74	60.3 (90.2)	-36.6 (-240.0 to 166.8)	0.69	96.9 (88.8)
**Large VLDL and chylomicron particles, nmol/L**									
Baseline, mean (SD)	8.8 (6.0)		7.8 (6.0)			8.2 (6.2)			8.8 (7.6)
Week 24	-9.6 (5.6)	-20.5 to 1.4	-4.3 (4.6)	-13.6 (-24.4 to -2.7)	0.0053	-3.0 (4.6)	-12.2 (-23.1 to -1.3)	0.012	9.2 (4.6)
Week 48	-11.8 (6.5)	-24.5 to 1.0	-4.5 (5.6)	-14.9 (-28.0 to -1.8)	0.011	-6.8 (5.7)	-17.2 (-30.5 to -3.9)	0.0038	10.4 (5.5)
**Medium HDL particles, umol/L**									
Baseline, mean (SD)	4.9 (4.2)		4.9 (3.6)			5.1 (4.3)			5.1 (4.6)

Week 24	157.4 (45.1)	68.8 to 245.9	173.8 (37.2)	92.5 (5.7 to 179.3)	0.017	163.4 (36.9)	82.1 (-5.5 to 170.0)	0.036	81.3 (36.6)
Week 48	165.1 (42.3)	82.1 to 248.1	191.3 (36.3)	79.9 (-3.4 to 163.3)	0.032	151.8 (36.5)	40.5 (-44.6 to 125.5)	0.29	111.3 (35.2)
**Medium VLDL particles, nmol/L**									
Baseline, mean (SD)	25.4 (24.4)		21.8 (25.6)			22.7 (26.1)			22.7 (22.3)
Week 24	18.9 (28.4)	-36.9 to 74.7	17.6 (23.9)	-59.6 (-115.0 to -4.1)	0.016	35.1 (23.6)	-42.1 (-97.8 to 13.6)	0.090	77.2 (23.7)
Week 48	-19.0 (86.5)	-188.8 to 150.8	-22.3 (75.9)	-194.3 (-369.8 to -18.9)	0.013	22.2 (76.0)	-149.9 (-328.5 to 28.8)	0.060	172.0 (73.9)
**Small HDL particles, umol/L**									
Baseline, mean (SD)	22.0 (5.7)		20.8 (5.2)			20.8 (5.4)			21.2 (5.5)
Week 24	-0.2 (2.9)	-5.9 to 5.5	0.7 (2.4)	-2.4 (-8.0 to 3.3)	0.35	-1.3 (2.4)	-4.4 (-10.1 to 1.3)	0.08	3.1 (2.4)
Week 48	3.8 (2.7)	-1.5 to 9.2	0.0 (2.4)	0.3 (-5.2 to 5.8)	0.91	-4.9 (2.4)	-4.6 (-10.2 to 1.0)	0.07	-0.3 (2.3)
**Small LDL particles, nmol/L**									
Baseline, mean (SD)	961.3 (356.9)		911.6 (311.6)			920.3 (316.2)			923.0 (334.0)
Week 24	-15.6 (3.5)	-22.5 to -8.7	-17.1 (2.9)	-13.2 (-20.0 to -6.3)	<0.0001	-15.8 (2.9)	-11.8 (-18.7 to -5.0)	0.0001	-4.0 (2.9)
Week 48	-24.5 (2.7)	-30.1 to -19.5	-18.3 (2.3)	-11.5 (-17.0 to -6.1)	<0.0001	-18.7 (2.4)	-11.9 (-17.5 to -6.4)	<0.0001	-6.8 (2.3)
**Small VLDL particles, nmol/L**									
Baseline, mean (SD)	27.8 (19.4)		23.4 (14.3)			24.7 (17.3)			23.8 (15.6)
Week 24	88.8 (35.3)	19.6 to 158.1	48.3 (29.4)	-11.6 (-80.0 to 56.8)	0.70	59.7 (29.3)	-0.1 (-69.2 to 68.9)	0.99	59.9 (29.3)

Week 48	79.6 (31.2)	18.4 to 140.8	35.1 (27.1)	-22.9 (-85.4 to 39.7)	0.41	68.1 (27.5)	10.2 (-53.9 to 74.3)	0.72	57.9 (26.8)
**VLDL and chylomicron particles, nmol/L**									
Baseline, mean (SD)	62.0 (37.1)		53.0 (33.9)			55.6 (37.0)			55.2 (30.6)
Week 24	-1.5 (6.0)	-13.3 to 10.2	-0.3 (5.0)	-18.7 (-30.3 to -7.0)	0.0003	8.5 (4.9)	-10.0 (-21.7 to 1.7)	0.056	18.5 (4.9)
Week 48	-6.4 (5.7)	-17.6 to 4.9	-7.6 (5.0)	-30.2 (-41.7 to -18.6)	<0.0001	1.6 (5.0)	-21.0 (-32.8 to -9.2)	<0.0001	22.6 (4.9)
**VLDL particle size, nm**									
Baseline, mean (SD)	55.4 (6.4)		55.7 (6.5)			55.5 (6.9)			56.3 (7.3)
Week 24	1.5 (1.1)	-0.7 to 3.6	0.4 (0.9)	1.1 (-1.1 to 3.2)	0.27	1.4 (0.9)	2.1 (-0.0 to 4.3)	0.028	-0.7 (0.9)
Week 48	1.1 (1.2)	-1.3 to 3.4	0.6 (1.0)	1.9 (-0.6 to 4.3)	0.085	0.2 (1.0)	1.4 (-1.0 to 3.9)	0.19	-1.2 (1.0)
**VLDL and chylomicron TG, mg/dL**									
Baseline, mean (SD)	122.1 (70.6)		108.5 (70.7)			112.5 (72.6)			118.1 (81.6)
Week 24	-13.4 (3.6)	-20.5 to -6.3	-10.4 (3.0)	-13.4 (-20.5 to -6.4)	<0.0001	-7.7 (3.0)	-10.7 (-17.8 to -3.6)	0.0007	3.0 (3.0)
Week 48	-16.4 (4.3)	-24.8 to -8.0	-14.7 (3.7)	-21.9 (-30.5 to -13.2)	<0.0001	-10.7 (3.7)	-17.8 (-26.5 to -9.0)	<0.0001	7.1 (3.6)

[a] Enhanced liver fibrosis (ELF) reported as change from baseline (CFB). All other endpoints reported as percent change from baseline (%CFB). Lipids, lipoprotein particles and lipoproteins are evaluated at Week 24 and Week 48 as LSM %CFB; other analytes as Week 52 LSM CFB.

CFB, change from baseline; CI, confidence interval; DB, double-blind; HDL, high-density lipoprotein; HDL-C, high-density lipoprotein cholesterol; IDL, intermediate-density lipoprotein; LDL, low-density lipoprotein; LSM, least squares mean; OL, open-label; RLP, remnant-like particle; SD, standard deviation; SE, standard error; VLDL, very low-density lipoprotein.
