# Supplementary material for: Resmetirom for nonalcoholic fatty liver disease: a randomized, double-blind, placebo-controlled phase 3 trial
Source: Nat Med. 2023 Oct 16;29(11):2919–28. doi: 10.1038/s41591-023-02603-1 (PMC10667098; doi:10.1038/s41591-023-02603-1)
Supplement: Supplementary file 2 — Reporting Summary [file 41591_2023_2603_MOESM2_ESM.pdf]

nature portfolio

Corresponding author(s): Stephen A. Harrison

Last updated by author(s): 5/31/2023

Reporting Summary

Nature Portfolio wishes to improve the reproducibility of the work that we publish. This form provides structure for consistency and transparency in reporting. For further information on Nature Portfolio policies, see our Editorial Policies and the Editorial Policy Checklist.

Statistics

For all statistical analyses, confirm that the following items are present in the figure legend, table legend, main text, or Methods section.

n/a	Confirmed	
☐	☒	The exact sample size (*n*) for each experimental group/condition, given as a discrete number and unit of measurement
☒	☐	A statement on whether measurements were taken from distinct samples or whether the same sample was measured repeatedly
☐	☒	The statistical test(s) used AND whether they are one- or two-sided<br>*Only common tests should be described solely by name; describe more complex techniques in the Methods section.*
☐	☒	A description of all covariates tested
☐	☒	A description of any assumptions or corrections, such as tests of normality and adjustment for multiple comparisons
☐	☒	A full description of the statistical parameters including central tendency (e.g. means) or other basic estimates (e.g. regression coefficient) AND variation (e.g. standard deviation) or associated estimates of uncertainty (e.g. confidence intervals)
☐	☒	For null hypothesis testing, the test statistic (e.g. *F*, *t*, *r*) with confidence intervals, effect sizes, degrees of freedom and *P* value noted<br>*Give P values as exact values whenever suitable.*
☒	☐	For Bayesian analysis, information on the choice of priors and Markov chain Monte Carlo settings
☐	☒	For hierarchical and complex designs, identification of the appropriate level for tests and full reporting of outcomes
☒	☐	Estimates of effect sizes (e.g. Cohen's *d*, Pearson's *r*), indicating how they were calculated

Our web collection on statistics for biologists contains articles on many of the points above.

Software and code

Policy information about availability of computer code

Data collection	No software was used
Data analysis	SAS

For manuscripts utilizing custom algorithms or software that are central to the research but not yet described in published literature, software must be made available to editors and reviewers. We strongly encourage code deposition in a community repository (e.g. GitHub). See the Nature Portfolio guidelines for submitting code & software for further information.

Data

Policy information about availability of data

All manuscripts must include a data availability statement. This statement should provide the following information, where applicable:

- Accession codes, unique identifiers, or web links for publicly available datasets
- A description of any restrictions on data availability
- For clinical datasets or third party data, please ensure that the statement adheres to our policy

Datasets generated as part of the MAESTRO-NAFLD-1 trial are considered commercially sensitive and as such, are not publically available. Requests for data supporting findings in this manuscript should be made to the corresponding author (S.A.H.). Data may be shared in the form of aggregate data summaries and via a data transfer agreement. Individual patient-level data is subject to patient privacy and cannot be shared.

Human research participants

Policy information about studies involving human research participants and Sex and Gender in Research.

Reporting on sex and gender	Sex was self-reported by the patient<br>Sex and/or gender was not considered in the design of the MAESTRO-NAFLD-1 trial and findings from the MAESTRO-NAFLD-1 trial do not apply to only one sex and/or gender
Population characteristics	Mean age, 56 years; female, 57%; white, 88%; Hispanic, 34%. High percentages of patients across all 4 arms had metabolic risk factors including obesity with mean BMI 35 kg/m2; type 2 diabetes, 49%; dyslipidemia, 88%; hypertension, 75%
Recruitment	Patients were recruited by referral and general advertisement for study participation
Ethics oversight	An institutional review board or independent ethics committee at each site approved the protocol and all amendments

Note that full information on the approval of the study protocol must also be provided in the manuscript.

Field-specific reporting

Please select the one below that is the best fit for your research. If you are not sure, read the appropriate sections before making your selection.

☒ Life sciences ☐ Behavioural & social sciences ☐ Ecological, evolutionary & environmental sciences

For a reference copy of the document with all sections, see nature.com/documents/nr-reporting-summary-flat.pdf

Life sciences study design

All studies must disclose on these points even when the disclosure is negative.

Sample size	Sample size was based on safety and regulatory considerations to facilitate the evaluation of the treatment effect within subgroups of interest. For evaluation of the key secondary endpoints, randomizing ≥200 patients to each of the 3 DB arms was expected to provide >90% power to demonstrate a statistically significant difference between each resmetirom dose and placebo at the two-sided 0.025 significance level in the percent change from baseline in LDL-C at Week 24, assuming a ≥13.5% difference between the resmetirom arm and placebo arm with a within-treatment standard deviation of 16%. Other key secondary lipid endpoints and percent change in hepatic fat between the resmetirom and placebo arms have ≥90% power. This trial was designed to maintain an overall study-wise type I error rate of α=0.05 for the key secondary endpoints only. The error rate was controlled by first splitting the overall two-sided α=0.05 into 2 partitions via the Bonferroni method, and then the key secondary endpoints tested in a prespecified hierarchical order
Data exclusions	No data were excluded from the analyses
Replication	Every analysis was performed twice for confirmation
Randomization	An interactive voice and web response system was used to assign treatment
Blinding	Patients and study personnel administering the study drug and performing the clinical assessments were blinded to individual patient's treatment (resmetirom or placebo). Select individuals were not blinded to individual patient's treatment (eg, to prepare data monitoring committee (DMC) materials); these individuals were not otherwise involved in the trial. Results of several laboratory tests (eg, lipids, SHBG, FT4) were blinded to study personnel and investigators during the trial to preserve the blind.

Reporting for specific materials, systems and methods

We require information from authors about some types of materials, experimental systems and methods used in many studies. Here, indicate whether each material, system or method listed is relevant to your study. If you are not sure if a list item applies to your research, read the appropriate section before selecting a response.

Materials & experimental systems

n/a	Involved in the study
☒	☐ Antibodies
☒	☐ Eukaryotic cell lines
☒	☐ Palaeontology and archaeology
☒	☐ Animals and other organisms
☐	☒ Clinical data
☒	☐ Dual use research of concern

Methods

n/a	Involved in the study
☒	☐ ChIP-seq
☒	☐ Flow cytometry
☒	☐ MRI-based neuroimaging

Clinical data

Policy information about clinical studies

All manuscripts should comply with the ICMJE guidelines for publication of clinical research and a completed CONSORT checklist must be included with all submissions.

Clinical trial registration	ClinicalTrials.gov identifier, NCT04197479
Study protocol	Not available
Data collection	MAESTRO-NAFLD-1 was conducted between December 16, 2019 and December 13, 2021 at 80 sites in the United States
Outcomes	The primary and secondary endpoints were predefined in the protocol and SAP based on clinically important endpoints for NASH clinical trials
